# Canonical and Alternative Auxin Signaling Systems in Mono-, Di-, and Tetraploid Potatoes

**DOI:** 10.3390/ijms241411408

**Published:** 2023-07-13

**Authors:** Sergey N. Lomin, Oksana O. Kolachevskaya, Dmitry V. Arkhipov, Georgy A. Romanov

**Affiliations:** Timiryazev Institute of Plant Physiology, Russian Academy of Sciences, Botanicheskaya 35, 127276 Moscow, Russia; losn1@yandex.ru (S.N.L.); vatrushbox@mail.ru (O.O.K.); hotdogue@yandex.ru (D.V.A.)

**Keywords:** gene expression, transcription regulation, auxin receptors, Aux/IAA proteins, ARF transcription factors, potato cultivars

## Abstract

It has long been known that the phytohormone auxin plays a promoting role in tuber formation and stress tolerance in potatoes. Our study aimed to identify and characterize the complete sets of auxin-related genes that presumably constitute the entire auxin signaling system in potato (*Solanum tuberosum* L.). The corresponding genes were retrieved from sequenced genomes of the doubled monoploid *S. tuberosum* DM1-3-516-R44 (DM) of the Phureja group, the heterozygous diploid line RH89-039-16 (RH), and the autotetraploid cultivar Otava. Both canonical and noncanonical auxin signaling pathways were considered. Phylogenetic and domain analyses of deduced proteins were supplemented by expression profiling and 3D molecular modeling. The canonical and ABP1-mediated pathways of auxin signaling appeared to be well conserved. The total number of potato genes/proteins presumably involved in canonical auxin signaling is 46 and 108 in monoploid DM and tetraploid Otava, respectively. Among the studied potatoes, spectra of expressed genes obviously associated with auxin signaling were partly cultivar-specific and quite different from analogous spectrum in Arabidopsis. Most of the noncanonical pathways found in Arabidopsis appeared to have low probability in potato. This was equally true for all cultivars used irrespective of their ploidy. Thus, some important features of the (noncanonical) auxin signaling pathways may be variable and species-specific.

## 1. Introduction

The tubers of potato (*Solanum tuberosum* L.) are well known as widespread sources of food, feed, and technical substances (starches). Auxin, a classical plant hormone, was consistently reported to promote potato tuber formation, in the study of both endogenous processes [1,2] and conventional in vitro systems with exogenous auxin [3,4]. Therefore, the auxin signaling system attracts constant interest of potato researchers [5,6,7].

Over the past decade, prominent progress has been achieved in potato research at the molecular level. This is due, first of all, to the sequencing of the complete genome of the *S. tuberosum* group Phureja doubled monoploid DM1-3 516 R44 (DM) by the Potato Genome Sequencing Consortium (PGSC) [8]. Afterwards, a set of genes/proteins controlling tuberization was uncovered [9,10]. It has now become clear that the regulation of tuberization is based on a complex crosstalk between numerous hormonal and non-hormonal factors [9,10,11,12,13].

In our research, we focused on the hormonal part of this regulatory network. On the basis of our experimental data [14,15,16,17] and data from recent literature, an updated hypothesis of hormonal regulation of potato tuberization at the molecular level was advanced [5,15], in which auxins play an important role, especially at the tuber induction and initiation stages.

*S. tuberosum* of the Phureja group is common in the highlands of Peru and Colombia, where it is represented by numerous varieties. Many varieties of Phureja are heterozygous diploids, but for breeding purposes, the generation of haploid lines was often followed by homozygous double monoploids (DMs) [18]. Comparison of Phureja haploid lines with cognate DM showed considerable similarity in most phenotypic traits (number and size of flowers and number of tubers) in each pair, although the main organs in DM were somewhat larger [19]. The tubers in DM R44 are strongly elongated spindle-shaped, although the commercial Phureja cultivars have traditional rounded tubers.

The DM R44 line proved to be a convenient model for sequencing the core potato genome, but the study of artificial lines does not provide a complete picture of the genetics, metabolism and tuber formation of real potato varieties with more complex genomes. Not surprisingly, the genome of the more productive heterozygous diploid *S. tuberosum* line RH89-039-16 (RH) was sequenced after the DM Phureja genome [20], followed by genomes of even more productive tetraploid potato varieties [21,22,23].

Receptors are key proteins in any hormone signaling; they are capable of high-affinity binding to the hormone and transmitting the signal to downstream target genes. For auxin, TRANSPORT INHIBITOR RESPONSE 1 (TIR1) and AUXIN SIGNALING F-boxes (AFB1–AFB5) are considered canonical receptors [24,25]. These proteins are localized mostly in the nucleus, although some of them (e.g., AFB1 of Arabidopsis) can reside in the cytoplasm and accomplish peculiar functions [25,26]. AUXIN BINDING PROTEIN 1 (ABP1), S-PHASE KINASE-ASSOCIATED PROTEIN 2A (SKP2A) and AUXIN RESPONSE FACTOR 3 (ARF3 or ETTIN, ETT) are now recognized as potential auxin receptors for alternative signaling in Arabidopsis [27,28,29]. The mechanism of auxin action involving F-box proteins (orthologs of TIR1/AFB proteins) as master receptors has been uncovered in the Arabidopsis model (for comprehensive reviews, see [30,31]). At low physiological concentrations of auxin, ARF transcription factors are inactive, being blocked by Aux/IAA repressor proteins. The latter bind TOPLESS/TOPLESS-RELATED (TPL/TPR) proteins, which interact with HDAC histone deacetylase, whose work renders chromatin transcriptionally inactive [32]. When auxin is added, the formed triple complex of auxin–receptor–Aux/IAA leads to the ubiquitination of the latter, involving SCF (SKP1–CUL1–F-box protein)–E3 ubiquitin ligase complex, followed by degradation of Aux/IAA in proteasomes [33,34]. The released transcription factors ARFs bind to the promoters of primary response genes. Consequently, auxin affects the expression of responsive genes by stimulating or suppressing the initiation of their transcription [35]. At the same time, *Aux/IAA* are some of the primary response genes, and their expression is activated by auxin. Thus, this system relies on negative feedback preventing the pathological overexpression of auxin-sensitive genes.

Studies in recent years have revealed that the canonical pathway is far from being the only system transducing the auxin signal from TIR1/AFB receptors [27,28,36,37].

As exemplified by Arabidopsis, many atypical Aux/IAA proteins lacking the DII domain (Degron) necessary for recognition by the auxin receptor/ubiquitination complex are nevertheless involved in auxin signaling. Being similar in structure to canonical Aux/IAA proteins, the “defective” proteins compete with them for binding to transcription factors [38]. In contrast to canonical IAA repressor proteins, whose degradation is promoted by auxin, atypical IAA repressors, on the contrary, are stabilized by auxin-induced phosphorylation. Thus, noncanonical Aux/IAA proteins interfere with canonical auxin signaling, reversing the sign of the effect at the level of gene expression. In Arabidopsis, such proteins include IAA32 and 34, which suppress cell growth, especially at high auxin concentrations [28,29].

Stabilizing phosphorylation of IAA32 and 34 is performed by the transmembrane protein kinase TMK1 initially localized on the plasma membrane. When the concentration of auxin is increased, the C-terminal catalytic fragment of TMK1 (possibly also of TMK4) splits off, translocates into the nucleus, and phosphorylates there the aforementioned Aux/IAA noncanonical proteins [39,40]. Such a model was confirmed by the auxin effect on the formation of the apical bend in seedlings [41].

Similarly, at the root tip, the noncanonical IAA33 protein is able to compete with typical Aux/IAA repressors for binding to ARF10,16 transcription factors, which also have abnormal structure. Elevated concentrations of auxin not only fail to promote IAA33 degradation in proteasomes but instead stabilize it by increasing the activity of Mitogen-activated Protein Kinase 14 (MPK14) [42,43]. The mechanism of the increased activity of auxin-responsive protein kinases caused by auxin remains to be addressed.

Two more noncanonical Aux/IAA proteins, IAA20 and 30, are involved along with the transcription factor ARF5/MONOPTEROS (ARF5/MP) in cellular differentiation of the root vascular system. The expression of these proteins is IAA-dependent, and the action of IAA20,30 is aimed at suppressing ARF5/MP activity [44,45]. MP has also been shown to interact with atypical IAA12/BODENLOS (BDL) at low auxin content.

Notably, all of the above examples are closely related to the canonical pathway of auxin signaling through the formation of the classical auxin–SCF^TIR1/AFB^–Aux/IAA complex, Aux/IAA repressor ubiquitination and degradation, and ARF transcription factor release and their effect on primary auxin response gene expression. The mentioned noncanonical Aux/IAA proteins only modulate (moderate) the action of this core auxin signaling module in one way or another.

However, at this time, real alternative mechanisms of auxin action, completely independent of the canonical pathway, have been discovered. One such pathway resembles the action of animal hormones and is based on the ability of the noncanonical transcription factor ARF3 (ETT, ETTIN) to bind auxin at high concentration, thus switching into an active state [32,46]. At low auxin concentrations, ARF3/ETT recruits HISTONE DEACETYLASE 19 (HDA19) and TPL proteins, which deacetylate and compact chromatin, suppressing the transcription of auxin-responsive genes [32]. Inflow of auxin and its binding to ARF3/ETT result in decay of the ARF3/ETT–TPL/HDA19 complex, providing a direct mechanism for chromatin decondensation and cognate genes activation. ARF3/ETT, TPL/TPR2, and HDA19 function cell-specifically to control gene expression during gynoecium development. Also, ARF3/ETT together with ARF4 take part in specifying leaf polarity by expressing asymmetrically on the abaxial side [47].

Very rapid cellular effects of auxin have been known for decades; for example, plasma membrane hyperpolarization, H^+^ fluxes in the plasma membrane, cytosolic Ca^2+^ transients, and protoplast swelling [37]. Another rapid, TIR1/AFB-independent effect of auxin is the regulation of the transport of PIN auxin transporters [48,49], although other authors consider this effect nonspecific [50]. This feedback of auxin to its own transport is the main prerequisite for so-called auxin channeling, a mechanism underlying self-organizing development, such as the formation of flexible vascular networks [51]. In addition, auxin very rapidly induces the phosphorylation of about a thousand proteins involved in various cellular functions in a TIR1/AFB-independent manner [52].

AUXIN BINDING PROTEIN 1 (ABP1), which already as of the early 1970s [53,54] was identified as an auxin-specific binding protein, is a candidate for mediating auxin entry into the TMK pathway, not least because of its association with TMK1 [55]. Arabidopsis ABP1 binds the natural auxin IAA at an acidic pH typical of the apoplast, where a small part of the ABP1 pool is secreted [27]. The ABP1–TMK1 signaling module on the cell surface is required for a rapid global phospho-response to auxin. *Abp1* and *tmk* mutants exhibit defects in cellular responses triggered by auxin, such as H^+^-ATPase activation, swelling of shoot protoplasts [56], and accelerated cytoplasmic movement. The marked dysregulation of protein phosphorylation observed in *abp1* and *tmk1-1* mutants suggests that ABP1 and TMK are key regulators of this process, reflected in some developmental defects in *abp1* and *tmk1-1* mutants grown under standard conditions [57,58]. Analysis of loss-of-function alleles and complementary lines showed that both ABP1 and TMK play a crucial role in auxin-initiated de novo formation and regeneration of the shoot vascular network, a classical result of auxin channelization, which is the mechanism underlying self-organizing plant development [59].

Another alternative mechanism is based on the properties of S-phase Kinase-associated Protein 2A (SKP2A), an F-box protein homologous to AFB auxin receptors and also localized in the nuclei [29,60,61]. This protein under the influence of auxin enhances the formation of specific protein associations in roots. These complexes become a substrate for protein ubiquitination similar to complexes with classical receptors. But the main targets for SKP2A are distinct transcription factors—E2FC and DPB—that are specific inhibitors of cell mitosis. The elimination of these proteins allows the cell to divide. Thus, intracellular auxin signaling in Arabidopsis is a complex set of biochemical reactions, interdependent or not, aimed mainly, but not only, at orchestrating the differential activity of genes. The intranuclear proteins ARF3 and SKP2A can be considered alternative auxin receptors, and, apparently, the number of auxin sensor proteins is not limited to them. At this stage, it is important to establish whether the recent schemes of noncanonical auxin signaling in Arabidopsis are equally suitable for describing this signaling in other plant species, including potato.

In the present work, a structural and functional study was carried out on the auxin signaling genes and proteins of potato of different genotypes: duplicated monoploid Phureja (DM), heterozygous diploid line *S. tuberosum* (RH), and commercial tetraploids Désirée and Otava, the genome of the latter also being fully sequenced [23]. The sets and structures of genes and proteins directly involved in auxin signaling were compared; active organ-specific genes were identified for each genotype. Previous studies in this direction have focused on StAux/IAA-type and/or StARF-type [62,63,64] genes/proteins. Somewhat greater coverage of auxin signaling elements was implemented in works [5,15,65] where potato receptors were investigated as well.

In contrast to these earlier works, the present genome-wide study aimed to cover as many elements of potato auxin signaling as possible, not only in the genetically simplified duplicated Phureja monoploid but also in heterozygous diploid and tetraploid potato lines. This large coverage enabled us to depict a plausible picture of complex auxin signaling using potato as a model plant. At the same time, we not only reproduced but also substantially revised the particular findings of previous studies. It is also important that, unlike previous works, we did not limit our study to canonical auxin signaling but also evaluated the possibility of different pathways of noncanonical (alternative) signaling of this phytohormone. Here, those genes/proteins are referred to as noncanonical that meet the criteria for participation in any of the noncanonical auxin signaling pathways. Our results demonstrate, along with features of similarity, certain differences in auxin signaling in potato compared to Arabidopsis, as well as cultivar-dependent variability in the expression patterns of auxin-responsive genes in potato itself, which may be the cause of genotype-dependent differences in tuber morphology as well as the productivity and stress tolerance of this crop.

## 2. Results and Discussion

### 2.1. Genes/Proteins Related to Canonical Auxin Signaling in Potato

**Figure 1:**
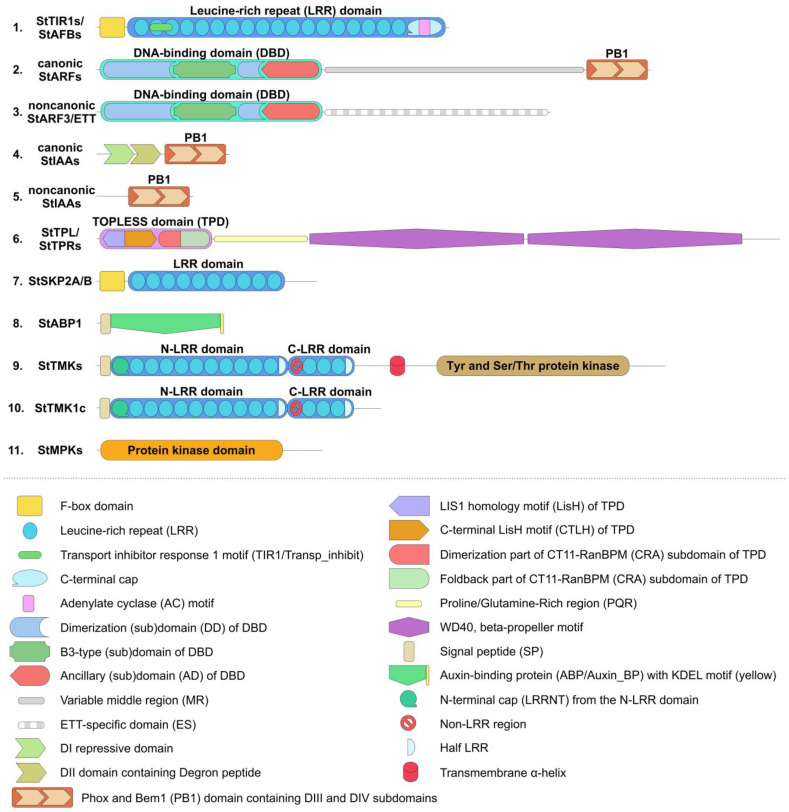
Overall structures and typical domain composition of auxin signaling-related proteins of potatoes. 1—StTIR1s, StAFBs—presumable auxin receptors. 2—StARF5, 6a, 6b, 8a, 8b, 8c, 19a, 19b, 19c—presumable transcription stimulators; StARF4; StARF1, 2a, 2b, 18a, 18b, 18c—presumable transcription inhibitors. 3—StARF3/ETT, putative noncanonical auxin receptors. 4—StAux/IAA1–4, 6–8, 10–12, 14, 15, 21–23, 25—presumable signal transduction inhibitors. 5—putative noncanonical StAux/IAAs. 6—StTPL, StTPRs—presumable transcription inhibitors. 7—StSKP2A/B—S-Phase Kinase-Associated Protein 2 homolog. 8—StABP1—presumable alternative auxin receptor. 9—StTMK1a, 1b, 4a, 4b, 4c, 5a, 5b, 5c—putative Ser/Thr protein kinases. 10—StTMK1c—TMK-lacking kinase domain. 11—StMPK1, 2—presumable MAP kinases.

The present study focused on proteins putatively involved in direct auxin signaling; enzymes for the synthesis or metabolism of auxin, as well as auxin transporters, were not considered. We confirmed the presence of five auxin receptor orthologs of the TIR1/AFB family in the haploid DM potato genome, as reported previously [5,15]. Their genes are arranged rather uniformly, with three exons encoding proteins 580–622 amino acid (aa) residues long (Figure 1 and Figure S1, Table S1). All presumable receptors have two domains in the protein structure. At the N-terminus, there is an F-box domain (PF18511.3) followed by a leucine-rich repeats (LRR)-containing domain consisting of 18 LRRs [66,67]. A specific unit of the LRR region is distinguished, located in the 2nd and part of the 3rd LR repeat, called the Transport inhibitor response 1 domain (Transp_inhibit) (PF18791.3), including an insertion of one short α-helix in the loop between the β-strand and the adjacent helix (see Section 2.4.1 for 3D structure details). An adenylate cyclase (AC) motif was detected at the C-terminus of the LRR domain of TIR/AFB [68] (Figure 1).

**Figure 2:**
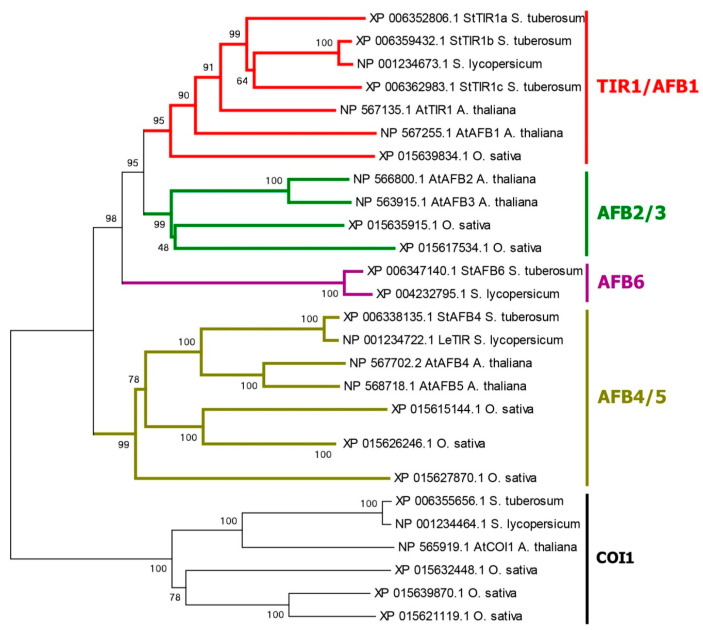
Phylogenetic analysis of TIR1/AFB auxin receptor orthologs. The protein sequences of potato, tomato, Arabidopsis, and rice were retrieved from NCBI database, and phylogenetic analysis was performed with MEGA6 program using neighbor-joining method. The percentage of replicate trees in which the associated proteins clustered together in the bootstrap test (1000 replicates) are shown next to the branches. The evolutionary distances were computed using the p-distance method. Gene classes are indicated with different colors.

However, the sets of receptor orthologs proved to be species-specific. DM potato has orthologs (StTIR1a, 1b, 1c) of AtTIR1 and AtAFB1 but no orthologs of AtAFB2,3 Arabidopsis receptors (Figure 2). In addition, potato possesses one ortholog of the AtAFB4,5 group, StAFB4, as well as a representative of a particular group, StAFB6. The latter group is missing in Arabidopsis. Rice, for example, has no orthologs of the latter group either and, unlike potato, has orthologs of AtAFB2,3 receptors. In potato varieties of greater ploidy, it is reasonable to expect higher gene redundancy compared to the DM cv. Phureja. Therefore, it was not surprising that in the recently sequenced genome of the tetraploid potato cv. Otava [23], as many as 13 canonical auxin receptor genes were identified (Table S1). Each DM gene corresponded to two or three of Otava’s orthologs structurally (by deduced aa sequence), proving almost identical to them.

We identified 24 *ARF* genes in the potato DM genome (Table S1), which is 2–4 genes more than the number published earlier [62,64]. The difference can be explained by the fact that the authors used the then-available annotation of the potato *Solanum tuberosum* genome in the Phytozome v4.03 database. In our work, we preferred to rely on the data from the NCBI database, which we consider more complete and convenient. Notably, the current annotation of the *Solanum tuberosum* genome in the Phytozome v6.1 database became significantly closer to that in NCBI.

**Figure 3:**
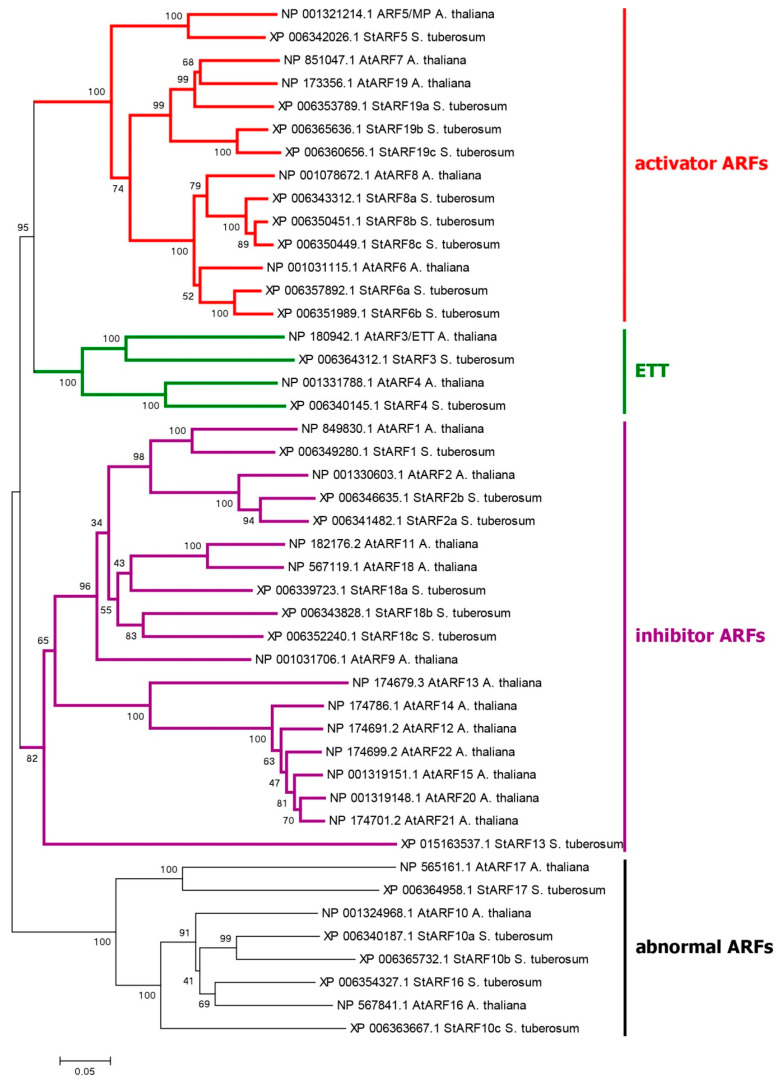
Phylogenetic analysis of ARF transcription regulator orthologs. The protein sequences of potato and Arabidopsis were retrieved from NCBI database, and phylogenetic analysis was performed with MEGA6 program using neighbor-joining method. For details, see legend to Figure 2 and Section 3.

ARF proteins can be functionally and phylogenetically divided into four clades (Figure 3). One of them unites presumable ARF activators, which form a distinct phylogenetic group. What is said above about the role of ARF in auxin signaling fits best for them. The corresponding genes consist of 13–14 exons encoding proteins 837–1114 aa long (Table S1). In general, these proteins include three main regions: at the N-terminus, the DBD (DNA-binding domain) (B3, PF02362.23, and Aux-respPF06507.15); in the middle, the non-regular MR (middle region, PF06507.15); and at the C-terminus, the PB1 (Phox and Bem1) domain (PF02309.18) (Figure 1 and Figure S2) [64,69] (see Section 2.4.3 for 3D structure details). The first region is responsible for interaction with promoters, and the last one is a protein–protein interaction domain (also termed the AUX_IAA domain, including DIII and DIV subdomains) mediating dimerization of ARFs and their binding with Aux/IAA proteins.

A variable middle region (MR) separates the ARF PB1 domain and the N-terminal DBD, which comprises a plant-specific B3-type (B3) subdomain, a dimerization (DD) subdomain, and an ancillary (AD) subdomain. The ability to activate or inhibit transcription is associated with some part of the MR [67,70].

As a result, we found nine functional activating ARFs in DM potato: StARF5, 6a, 6b, 8a, 8b, 8c, 19a, 19b, and 19c (Figure S2). In Otava tetraploid, 56 *ARF* genes were detected, out of which 20 belong to a similar group. All members of this group share a canonical structure (Figure 1).

The second clade is formed by presumable ARF inhibitors, which suppress the expression of target genes. This group includes seven representatives in DM potato. These genes consist of 14 exons encoding functional 627–845 aa proteins. They generally have the same structure of three domains as ARF activators (Figure 1 and Figure S2). However, one of the proteins, StARF13, has significant abnormalities in all domains. Accordingly, there remain six fully functional ARFs of this group: StARF1, 2a, 2b, 18a, 18b, and 18c. In potato cv. Otava, we found 12 ARFs of this group, 11 of which share the canonical structure (Table S1).

The third small clade includes orthologs of the *AtARF3* gene, encoding ortholog(s) of the ARF3/ETT protein, which was reported to explore a separate noncanonical pathway for auxin signaling [32,46]. This clade is discussed below in the respective section.

The remaining ARF proteins have little chance of being auxin-related, since none of them has structural details necessary to participate in either type of auxin signaling. For example, StARF10a, 10c, and 17 have strong lesions in the PB1 domain, and StARF11 has nothing but a single PB1 domain at the C-terminus (Figure S2). DM cultivar has 6 such ARFs, whereas Otava has 17 (Table S1). As there is no evidence of their participation in signal transmission, such genes/proteins were of little interest for our study.

**Figure 4:**
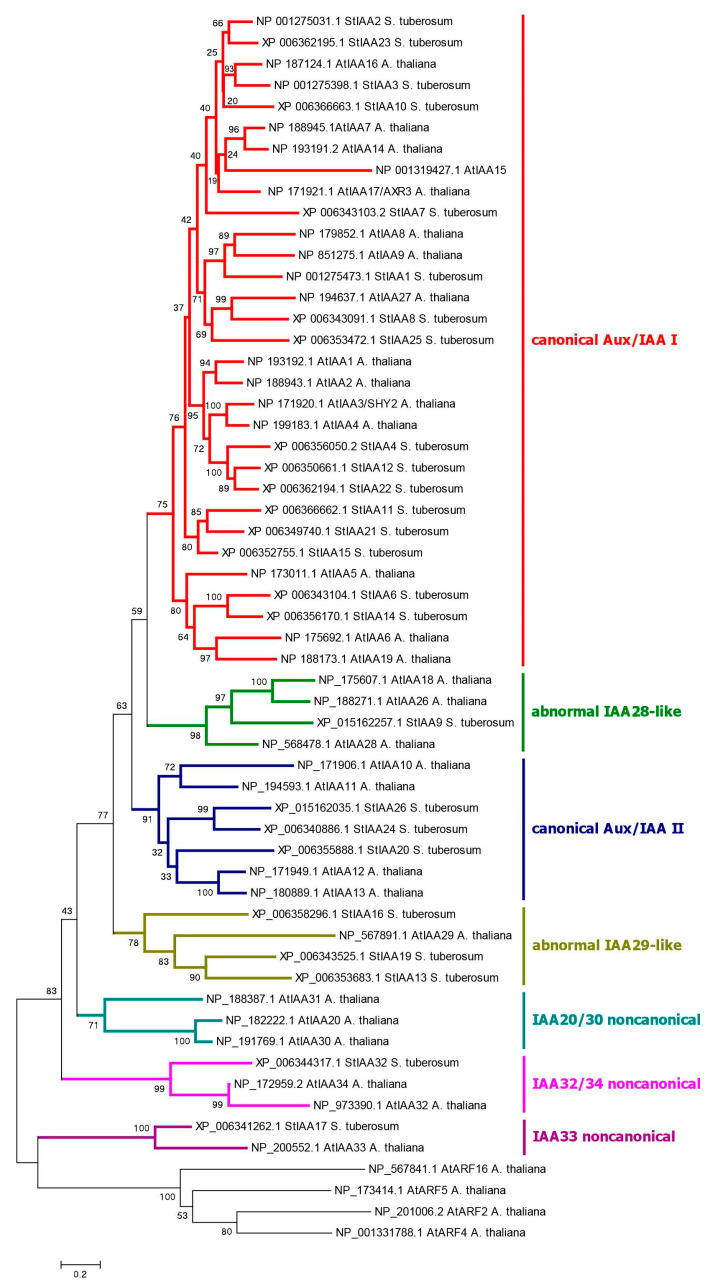
Phylogenetic analysis of Aux/IAA repressors of auxin signaling. The protein sequences of potato and Arabidopsis were retrieved from NCBI database, and phylogenetic analysis was performed with MEGA6 program using neighbor-joining method. The evolutionary distances were computed using the equal input method. For details, see legend to Figure 2 and Section 3.

Finally, the third component of the canonical auxin signaling pathway, Aux/IAA proteins, is encoded by 25 orthologous genes in DM potato (Figure 4), which is one fewer gene than previously reported [63]. This difference is due to the fact that the *StIAA5* and *StIAA18* genes are not annotated in NCBI, but there is an additional gene that we denoted as *StIAA32* because of its similarity to the Arabidopsis *AtIAA32* gene. In general, the *StAux/IAA* genes have three to six exons, and the deduced proteins are relatively short, ranging in size from 143 (StIAA17) to 349 (StIAA1) aa (Figure 1). They contain three main domains: DI (EAR, Ethylene-responsive element binding factor-Associated Repressor), DII (Degron), and PB1 (homologous to the ARF proteins domain of the same name) [71]. The latter domain is responsible for the interaction of these proteins with ARFs and among themselves. DI, aka EAR, contains the LxLxL motif, which provides interaction with TPL/TPR inhibitor proteins. Six potato Aux/IAAs lack this motif: StIAA13, 16, 17, 19, 26, and 32 (Figure S3).

The DII domain is of particular interest. It has the second name “Degron” and is involved in auxin binding. Accordingly, it generally determines whether a given IAA-related protein is able to participate in canonical auxin signaling, i.e., interacts with the hormone, with subsequent ubiquitin-mediated degradation in proteasomes. It was found that DII must contain the QVVGWPPV/IRxxR motif to be effective [33]. Altogether, 16 potato Aux/IAA repressors do possess such a conserved motif: StIAA1–4, 6–8, 10–12, 14, 15, 21–23, 25 (Figure 1; Table S1). All of them are homologs and form a cluster on the phylogenetic tree designated as canonical IAAs (Figure 4).

The PB1 domains include the so-called DIII and DIV subdomains (Figure 1 and Figure S3). The DIII subdomain contains a conserved lysine, and the DIV subdomain contains an OPCA-like motif, which are essential for protein-binding function [71,72]. In potato, only StIAA17 lacks a conserved lysine, while the OPCA-like motif is detected in all members of the family.

The above characteristics of *Aux*/*IAA* genes/proteins have been in part described previously [62,63]. We identified as many as 47 canonical *StIAA*s in the Otava genome, with a total number of *Aux*/*IAA* genes equal to 56 (Table S1). It is worth noting that the StIAA3 ortholog in Otava is annotated as having a rather unusual structure. Its PB1 domain is followed by two additional domains, Prefoldin_2 (PF01920.23) and TatA_B_E (PF02416.19). We performed a special investigation, which has shown that the emergence of such a complex gene may be associated with the fusion of the *StIAA3* gene with two neighboring genes, LOC102582738 (probable prefoldin subunit 4) and LOC102583072 (sec-independent translocase protein TATA, chloroplastic). Therefore, artifactual gene fusion in the processes of gene library creation, DNA sequencing, and/or gene annotation cannot be ruled out.

There remain six more StIAA proteins in DM potato (Table S1). Their genes have two to six exons encoding proteins ranging from aa 143 to 322. Interestingly, tetraploid Otava demonstrates less diversity in terms of Aux/IAA orthologs than DM potato (Figure 4). Thus, Otava has no StIAA13,17 orthologs, and the StIAA19 ortholog is truncated up to 79 aa. StIAA26 orthologs are annotated in cv. Otava has an unusual structure: upstream of the PB1 domain, it harbors a C2 domain (PF00168.33), which is characteristic of calcium-binding proteins. As described above, this appears to result from a fusion with a corresponding neighboring gene. Therefore, the artificial origin of such a pseudo locus, which does not exist in nature, is likely. But even so, this shows a possible pathway for the formation of complex multidomain proteins during evolution due to deletions of intergenic spacers in chromosomes.

**Figure 5:**
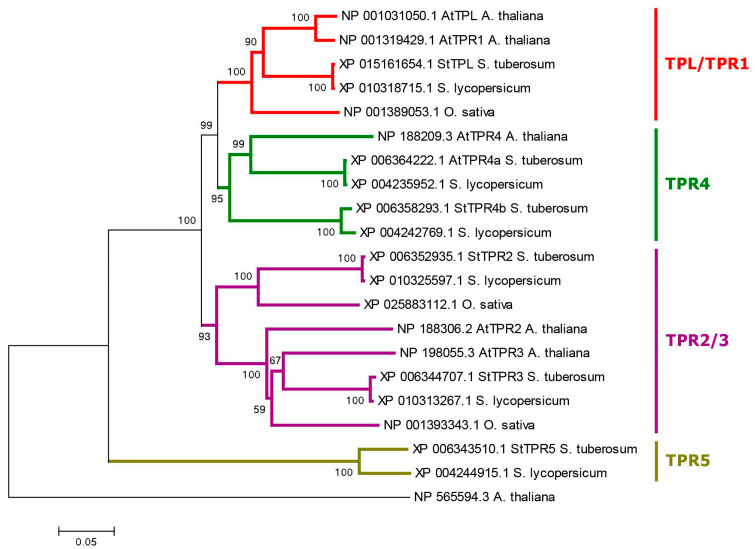
Phylogenetic analysis of TPL/TPR chromatin repressors. The protein sequences of potato, tomato, rice, and Arabidopsis were retrieved from NCBI database, and phylogenetic analysis was performed with MEGA6 program using neighbor-joining method. For details, see legend to Figure 2 and Section 3.

The *TPL*/*TPR* family is represented in DM potato by 6 gene orthologs (Table S1). The encoded proteins are rather large, ranging in length from 1132 (StTPL) to 1180 (StTPR5c) aa. They are contained in the N-terminus TOPLESS domain (TPD), including LISH-motif (subdomain) and CT11-RanBPM (CRA) subdomains. Within the latter, two parts are distinguished: dimerization and foldback [73]. At the C-terminus, there are two WD40 domains consisting of numerous WD40 repeats forming a beta-propeller structure (Figure 1) [74] (see Section 2.4.3 for 3D structure details). Phylogenetically, this family generates four groups, among which TPR5 orthologs are specific to the genus *Solanum*, as they are missing in Arabidopsis and rice (Figure 5). The genes of this family include a large number of exons, from 24 to 32 per gene. Tetraploid Otava has 14 *TPL*/*TPR* genes.

### 2.2. Genes/Proteins for Noncanonical Auxin Signaling in Potato

To date, much data on noncanonical auxin signaling have been accumulated, with Arabidopsis as a model plant. Here, we present the results of our search for homologous genes in potato plants.

One long-known candidate for plasma-membrane-affecting auxin receptors is the small protein ABP1 [53,54]. DM potato has one gene, *ABP1*, encoding a 202 aa protein (Table S1). The gene consists of five exons. One domain is present, which is identified as Auxin Binding Protein (PF02041.19, Auxin_BP) and belongs to the Cupin domain superfamily (PF07883.14) (Figure 1). The N-terminus has a hydrophobic site and a signal peptide directing to the ER, while the C-terminus has a KDEL-sequence characteristic of soluble ER resident proteins (see Section 2.4.2 for 3D structure details). There are four *ABP1* genes in the Otava variety (Table S1). Two of them encode proteins 207 and 223 aa in length, while the other two encode shorter 164 aa proteins. The short versions lack signal peptide, but all have the KDEL sequence. According to recent studies with Arabidopsis, ABP1 acts in concert with TMK-family protein kinases, in particular TMK1 and, possibly, also TMK4 [27]. Therefore, it was of interest to examine this family in more detail. In DM potatoes, the *TMK* family is represented by nine genes (Table S1). At the N-terminus, these proteins contain two LRR domains separated by a non-LRR fragment. The N-terminal (upstream) LRR domain contains LRRNT (N-terminal cap), 10 complete LR repeats, and 1 LRR fragment, while the downstream LRR domain contains 3 complete and 1 incomplete LRR (Figure 1) [75]. The Protein Tyrosine and Serine/Threonine Kinase (PF07714.20) catalytic domain resides at the C-terminus of TMKs (Figure 1) (see Section 2.4.3 for 3D structure details).

**Figure 6:**
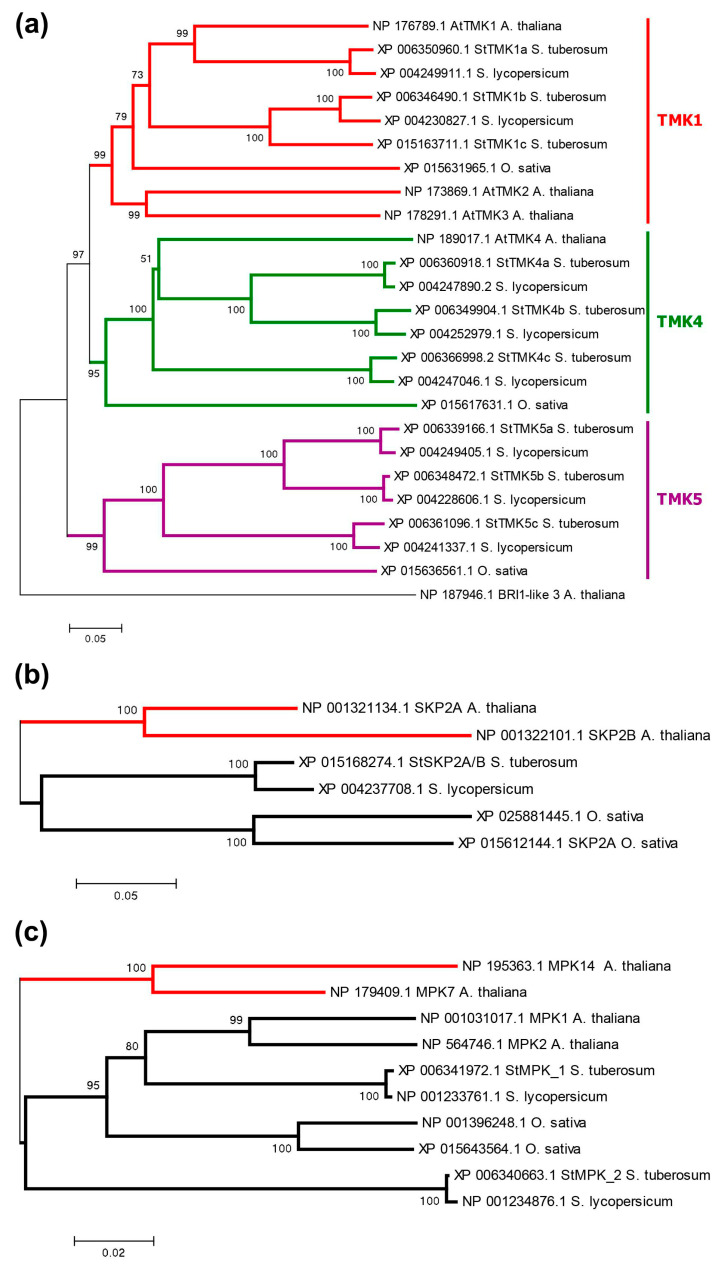
Phylogenetic analysis of proteins presumably involved in noncanonical auxin signaling. The protein sequences of potato, tomato, rice, and Arabidopsis were retrieved from NCBI database, and phylogenetic analysis was performed with MEGA6 program using neighbor-joining method. For details, see legend to Figure 2 and Section 3. (**a**). TMK1-like protein kinases. (**b**). SKP2 F-box proteins. (**c**). MPK protein kinases.

Phylogenetically, potato TMKs are divided into three groups (Figure 6a), represented in DM by StTMK1, 4, and 5. Surprisingly, there are no representatives of the latter in Arabidopsis, although they are present in tomato and rice. Each potato group contains three representatives; in the first group, StTMK1c appears to be defective, lacking TM and kinase domains. All other TMKs share a typical structure (Figure 1 and Figure S4), so altogether, 8 TMKs seem to be functional in DM potato. The genes consist of two exons, and protein lengths range from 932 to 978 aa (Table S1). Tetraploid Otava has 23 *TMK* genes, out of which 2 encode truncated orthologs of StTMK4c that lack crucial domains. Therefore, 21 *TMK* genes are considered functional in cv. Otava.

An important group of candidates for the role of noncanonical auxin signaling factors are the AtETT/ARF3 orthologs. For AtARF3, the interaction with auxin has been demonstrated directly [76]. DM potato has two members of this family, StARF3 and StARF4. These two proteins are close relatives (Figure 3), although they have obvious differences. In ARF3 orthologs, the structure of PB1 domains is drastically altered. In contrast, StARF4 and AtARF4 apparently possess functional PB1 domains, which indicates the possibility of their participation in canonical auxin signaling (see Section 2.4.3 for 3D structure details). The genes in this group have 12 coding exons, and the deduced protein length ranges from 747 to 811 aa (Table S1). In potato Otava, we identified seven genes of this group. Like the DM genes, the Otava *ARF3* orthologs lack the PB1 domain, whereas the *ARF4* orthologs contain it.

The F-box protein SKP2A of Arabidopsis in the presence of auxin promotes cell division by eliminating S-phase inhibitors of the cell cycle via ubiquitin-mediated degradation in proteasomes [29,60]. As for potato, it has no close ortholog of this gene, but it does have a common ortholog to the *AtSKP2A* and *B* genes, termed *StSKP2A/B* (Figure 6b).

This gene consists of five exons, of which four encode the 365 aa protein. The structure of SKP2A/B includes an F-box domain and an LRR domain. The latter contains fewer LR repeats and is significantly shorter than the analogous domain in TIR/AFB (Figure 1). SKP2A from Arabidopsis has similar structure (see Section 2.4.3 for 3D structure details). The Otava variety has two *StSKP2A*/*B* genes (Table S1). Nevertheless, the lack of a direct ortholog of SKP2A calls into question the functioning of this signaling mechanism in potato. This doubt is reinforced by the fact that this noncanonical path has been proposed by only one research team and, as we know, there has been no independent confirmation since the first publication [60] 15 years ago.

One noncanonical pathway modulating the core auxin signaling in Arabidopsis involves the repressor protein AtIAA33 and the stabilizing protein kinase AtMPK14 [29,43]. As it turned out, potato has no close orthologs of the MPK14 protein kinase. The two closest proteins are homologous to a large group of Arabidopsis kinases, including AtMPK1, 2, 7, and 14 (Figure 6c). These quasi-orthologs have been termed StMPK1 and StMPK2. Their genes consist of three exons, of which two encode proteins. These proteins are 370–372 aa in length and include the protein kinase domain (Pkinase, PF00069.28) (Figure 1) (see Section 2.4.3 for 3D structure details). These structural features correspond to the Arabidopsis orthologs. Otava has four representatives of this group, among which StMPK1 orthologs are shortened in size (234 and 264 aa) and therefore may be nonfunctional (Table S1). The lack of genuine homology between Arabidopsis MPK14 and its closest potato orthologs also casts doubt on the functioning of this mechanism in potato. This doubt is further reinforced when considering the second component of this signaling pathway, namely, the ortholog of the AtIAA33 protein. Potato, indeed, has such a close gene ortholog, namely *StIAA17* (Figure 4), lacking DI and Degron domains (Figure S3). However, as noted above, the potato variety Otava lacks the *StIAA17* gene (Table S1), though that evidently does not impair development and yield of these plants.

Phylogenetic analysis of Aux/IAAs in potato shows that there are no direct orthologs for the IAA20,30→MP pathway in potato. In contrast, potato has a close ortholog of AtIAA32, which interacts in Arabidopsis with ARF5/MP transcription factor (Figure 3 and Figure S2) [77]. Most close in structure is StIAA16, which lacks a DI domain but has a functional DII domain (Degron), so it should participate in canonical rather than noncanonical signaling. Therefore, there are doubts about the occurrence of this noncanonical pathway, too.

### 2.3. Expression Patterns of Auxin-Related Genes

#### 2.3.1. Canonical Genes

We performed a comparative analysis of expression patterns of auxin-signaling genes in three potato ecotypes (cultivars) differing in genome ploidy: the already mentioned lines of duplicated monoploid DM and heterozygous diploid RH, as well as commercial tetraploid cultivar Désirée. Gene expression data for the first two ecotypes were retrieved from the transcriptome database, which included all spectra of splice forms of cognate transcripts (see Section 3). Gene expression data for the potato cultivar Désirée were obtained experimentally by RT_qPCR. Due to the difference in methods, we will compare not so much the absolute values of individual gene expression as its global patterns.

**Figure 7:**
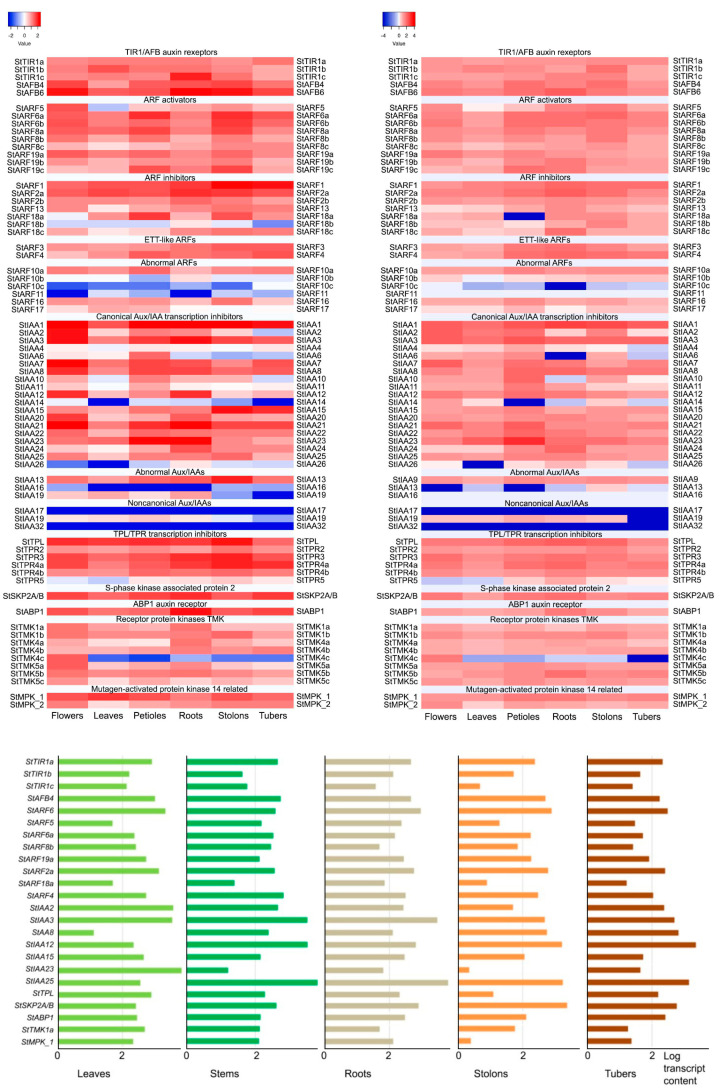
Organ-specific transcriptome profiling in potato cultivars of different ploidy. **Upper** panel: left, DM potato; right, RH potato. **Lower** panel: comparison of gene expression patterns in potato cv. Désirée.

Let us begin by considering canonical signaling (Figure 7; Figures S5 and S6; Table S1). As indicated above, a total of 40 genes of the haploid potato genome with all the necessary functional domains can directly participate in this signaling. Encoded are 5 TIR1/AFB-type receptors, 16 ARF-type transcription factors, and 16 Aux/IAA-type inhibitors of these factors. Quantitatively, the indicated set of genes is quite comparable to that in Arabidopsis [5]. In the tetraploid cultivar, the number of such genes in the minimal genome expectably increases and reaches, in the example of Otava cultivar, 94 units (excluding *TPL/TPR* genes).

Among the presumable transcription activators, StARF6a and StARF6b seemed to be the most versatile. The cognate genes were strongly expressed in all three ecotypes and in all organs (Figure 7). However, *StARF8a* and *StARF19a* expression dominated in leaves of DM and RH potatoes. *StARF5* was strongly expressed in DM and RH flowers and to a lesser extent in stolons, but it was weakly expressed in petioles and especially leaves of all cultivars. It is worth noting that a relatively high level of *StARF5* expression in the RH ecotype was found in flowers, roots, and stolons, whereas in cv. Désirée, the expression of this gene was noticeable primarily in roots and tubers. In contrast, *StARF8b* exhibited in Désirée a higher level of expression in aerial than in underground organs. At the same time, in DM and RH potatoes, the enhanced level of common ARF activator expression was found in flowers, petioles, and stolons. In general, due to the foregoing differences, the specificity of StARF activators with respect to auxin-responsive target genes can be manifested [78].

According to transcriptome profiling, the main negative regulators in all organs of all ecotypes appeared to be StARF2a and possibly also StARF1 (Figure 7). However, they were supplemented in DM potato by StARF18a in petioles and stolons, as well as by StARF18c in underground organs, and in RH variety by StARF18c in roots and StARF18b in stolons. Apart from the latter, *StARF18b* was the rarest in the expression events among repressive transcription factors.

Among Aux/IAA repressor orthologs, StIAA1 appeared to be the most active and versatile factor, since the cognate gene was prominent almost everywhere (Figure 7). *StIAA3*, *8*, *21* and *23* were slightly inferior to it in versatility, prevailing in expression rate in several cases. *StIAA2* gene expression was detected in all organs of cv. RH, whereas in DM potato, this gene was silent in tubers. For some genes, transcripts were almost absent (*StIAA14*) or detected at low level (*StIAA6*, *10*, *11*, except petioles) in DM potato. In RH cultivar, low expression was noticed for *StIAA4* (except petioles) and *StIAA14* (except flowers). Several genes (*StIAA1*, *3*, *8*, *15*, *23*) were equally active in stolons and tubers of all ecotypes, while some others (*StIAA2*, *10*, *23*, *25*) exhibited a decreased activity in tubers compared to stolons. Such genes may be envisaged to participate in stolon growth and tuber emergence.

Since TPL/TPR proteins are involved in bringing the auxin signal to the target genes [74], we studied the expression of the corresponding orthologous genes (*StTPL/TPR2*, *3*, *4a*, *4b*, *5*) in double monoploid (DM) and heterozygous diploid (RH) potato (Figure 7). It turned out that the level of *StTPL* gene transcription was quite high in all organs of both DM and RH varieties, and in almost all cases, this gene was leading in expression in its group. Among the *StTPR* genes, two, *StTPR3* and *StTPR4a*, were actively expressed at close to *StTPL* levels; the other two, *StTPR2* and *StTPR4b*, were expressed more weakly but in all organs; and the fifth, *StTPR5*, was almost unexpressed in flowers and leaves and very weakly expressed in tubers.

#### 2.3.2. Noncanonical Genes

The ortholog of one of the most known alternative auxin receptors, StABP1, was quite strongly expressed in a constitutive-like mode, i.e., similarly extensive in different organs (Figure 7, Figures S5 and S6). This was equally true for each of the potato cultivars under study, regardless of their ploidy.

Expression of the *StTMK* transmembrane kinase family genes whose products are expected to interact with the ABP1 protein showed both common features and some distinctions between DM and RH ecotypes (Figure 7). In both cultivars, the maximum *TMK* expression occurred in flowers. In particular, *StTMK4c* was highly expressed only in flowers and virtually nothing else, and it was expressed more strictly in DM potatoes. In contrast, both orthologs of Arabidopsis *TMK1*, *StTMK1a*, and *StTMK1b* were highly or at least moderately expressed in all tested organs of both cultivars. Overall, the extensive expression of these and other StTMK kinases and the availability of expressed StABP1 support the participation of these proteins in a noncanonical auxin signaling pathway in potato.

Another possible noncanonical auxin signaling pathway would be through *ARF3/ETT* transcription factor orthologs. In Arabidopsis, the *ARF3/ETT* gene product is able to bind auxin directly and activate primary response genes [46,76]. A close ortholog of this gene, *StARF3*, is present in the haploid potato genome. It is markedly expressed in all organs of DM and RH potato (Figure 7). Interestingly, a close paralog of StARF3, StARF4, has canonical ARF protein structure. Thereby, it seems to participate the most probably in canonical rather than in alternative auxin signaling.

One more potential auxin signaling enhancer that is relatively close to, although not a direct ortholog of, Arabidopsis SKP2A, which can directly bind auxin, is StSKP2A/B. Its coding gene was strongly expressed in each organ of all studied cultivars (Figure 7). The highest level of expression of this gene in DM was observed in stolons and tubers, and in RH, expression was highest in roots, slightly lower in flowers and petioles of both ecotypes, and the lowest in leaves. In cv. Désirée, the expression level of *StSKP2A/B* was fairly high in most organs, with a maximum in stolons and a minimum in leaves.

Since the mechanism of suppression of the excessive auxin response by TMK1-mediated phosphorylation of the noncanonical repressors IAA20,30 and IAA32, 33, 34 has also been described [39,42,44,45], we analyzed the expression of orthologs of these genes in potatoes.

Phosphorylation of IAA33 repressors in Arabidopsis involves the phosphokinase AtMPK14, which transfers phosphate from TMK1 to IAA33. The closest but indirect orthologs of the respective gene in the potato genome, *StMPK1* and *StMPK2*, were active in all DM and RH organs. *StMPK1* was expressed at a higher level, and *StMPK2* was mainly moderately expressed in all organs except leaves, where the expression decreased (Figure 7). Nevertheless, the functioning of this signaling pathway in potato is questionable, primarily because of the absence of close homology between StMPK1 or StMPK2 and AtMPK14 (Figure 6c). On the other hand, there are also problems with the Aux/IAA repressor gene encoding a putative partner of these kinases. As noted above, in the potato genome, the closest ortholog of implicated *AtIAA33* is the *StIAA17* gene (Figure 4). However, in DM and RH varieties, the expression level of this gene is almost zero (Figure 7), which is consistent with the lack of this gene in cv. Otava. Consequently, the obtained data do not support the existence of this noncanonical pathway for auxin signaling modulation in potato.

As for the other known noncanonical Aux/IAA repressors in Arabidopsis, the expression data provide evidence not for but rather against the existence of similar pathways in potato. Potato has no direct orthologs for *IAA20*, *30* (Figure 4). Among other repressors, *StIAA13*, *16*, *19* are among the closest homologs, with *StIAA13* and *StIAA16* being almost unexpressed in DM and RH potato, respectively. *StIAA19* was silent in tubers of both low-ploidy ecotypes (Figure 7). As regards noncanonical *AtIAA32,34* genes, their closest ortholog does exist in potato: the *StIAA32* gene (Figure 4). However, *StIAA32* expression appeared to be at the background level in all DM and RH organs and very close to it, at the edge of device sensitivity, in cv. Désirée (Figure 7). On the other hand, many of the *Aux/IAA* genes in Arabidopsis [71] as well as potato [63,65] can become upregulated under conditions of elevated auxin content. Thus, *StIAA32* gene may be functional despite the low expression level registered in our work.

In general, with a few exceptions, the expression levels of some of the studied genes for putative noncanonical auxin signaling in potato were not inferior to the expression of genes for the main signaling module TIR1/AFB-ARF-IAA (Figure 7). The functioning mode of some of these noncanonical genes is still unclear. Therefore, it cannot be ruled out that potato may explore species-specific pathways of noncanonical auxin signaling that are absent in Arabidopsis. The study of unexpressed or low-expression noncanonical signaling genes has also raised questions. Whether this indicates permanent blocking of the putative signaling paths that may be associated with the aforementioned genes, or whether they simply need specific inductors to start expressing, remains to be addressed.

#### 2.3.3. Quantitative Comparison of Expression Patterns of Auxin-Signaling Genes

To evaluate more quantitatively the similarity degree among expression patterns of auxin signaling genes in potato ecotypes, the pairwise correlation coefficients (CC) according to Spearman’s algorithm were determined (Table S2). Correlation analysis showed a large pattern similarity between DM monoploid and RH diploid, with CC ranging from 0.662 (stolons) to 0.813 (tubers). It was of interest to compare separately CC of genes belonging to canonical auxin signaling and genes of alternative pathways. This analysis was performed (Table S2) and showed a trend towards higher correlation between noncanonical genes compared to canonical ones.

This result can be explained by the much higher redundancy of core auxin signaling genes, which encode multiple interchangeable proteins. On the contrary, noncanonical signaling pathways, as a rule, are built on low-redundancy elements, and if they are involved in vital processes, intervarietal correlations have to be stronger, which is what we observe de facto. Overall, this analysis provides additional evidence for the presence and importance of noncanonical auxin signaling pathways in potato. Thus, despite certain intervarietal differences exemplified by DM and RH ecotypes, we can conclude that general expression patterns of most auxin signaling genes in different potato ecotypes are similar.

### 2.4. Molecular Modeling of Key Elements of Auxin Signaling

In silico data are increasingly being used to test hypotheses related to protein function, including signaling. We applied molecular modeling techniques and docking to further assess the functionality of key auxin signaling proteins in potato.

#### 2.4.1. Modeling of 3D Structure of Canonical Auxin Receptors of TIR1 Superfamily

Models of all five potato auxin receptors were generated by homology modeling: StTIR1a, StTIR1b, StTIR1c, StAFB4, and StAFB6. A model of each of the receptors was built in complex with the StSKP1b protein, the peptide fragment of the Aux/IAA23 protein, and the inositol-6-phosphate (IP6) molecule; each complex was obtained in three versions differing in ligand: indolyl-3-acetic acid (IAA), naphthaleneacetic acid (1-NAA), and 2,4-dichlorophenoxyacetic acid (2,4-D) (15 variants in total).

**Figure 8:**
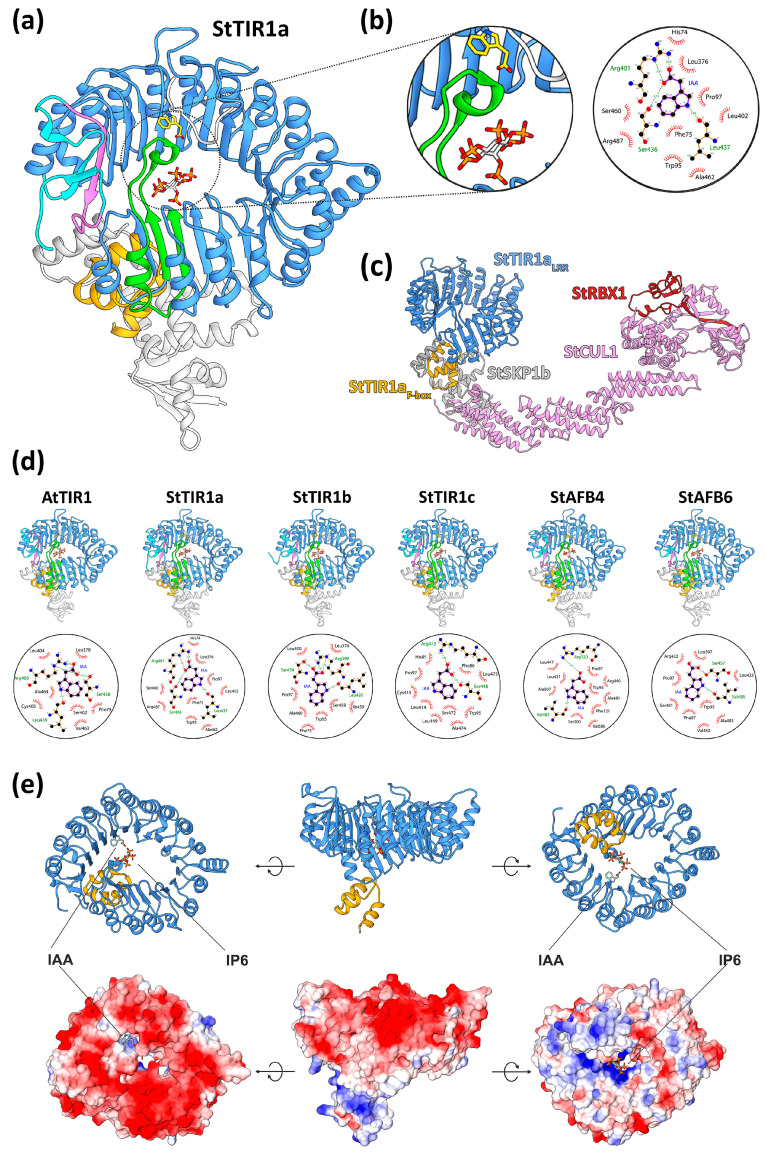
Modeling of 3D structure of canonical auxin receptors of TIR1 family: (**a**) Model of the spatial structure of canonical potato receptor StTIR1a in complex with IAA and IP6 ligands, StSKP1b protein (marked grey), and a peptide fragment of StAux/IAA23 protein. The F-box site of TIR1 receptor is stained yellow, the LRR domain is stained blue. Transport inhibitor response 1 motif (TIR1/Transp_inhibit) is highlighted in green; C-terminal cap is marked in cyan; adenylate cyclase (AC) motif is marked in magenta. In the ligands, oxygen atoms are colored red, nitrogen atoms are blue, phosphorus atoms are orange, carbon atoms are colored yellow in IAA and gray in IP6. (**b**) IAA- and IP6-binding sites in StTIR1a are highlighted in the left circle; the right circle shows the IAA binding diagram obtained in LigPlot. (**c**) Molecular model of potato SCF complex, obtained using AlphaFold multimer. StTIR1a (LRR domain)—blue; StTIR1a (F-box domain)—yellow; StSKP1b—grey; StCUL1—pink; StRBX1—red. (**d**) Models of the spatial structure of canonical potato TIR1 family auxin receptors (in complex with same ligands and proteins as StTIR1a), compared with the crystal structure of the Arabidopsis AtTIR1 receptor complex with the same ligands, the AtSKP1a protein (marked grey), and the peptide fragment of the AtAux/IAA7 protein (PDB ID: 2P1Q) [66]. Color scheme is the same as in (**a**) block; lower circles show the IAA binding diagrams obtained in LigPlot. (**e**) Homology model of the StTIR1a receptor (in three projections) in complex with IAA and IP6. Illustrated representation (**top**): blue color indicates the LRR domain, yellow indicates the F-box domain. Electrostatic potential of the molecular surface (**bottom**). Red—negative charge, blue—positive charge, white—neutral zones.

The expected structural and functional similarity of potato StTIR receptors with Arabidopsis TIR1 was shown (Figure 8a,b,d). The canonical receptors of the TIR1 superfamily include two structural subdomains: the first is a F-box subdomain, consisting of three α-helices, and the second is the leucine-rich repeat (LRR) domain, which has an α/β horseshoe fold and belongs to the solenoid protein domain type. The structure of the LRR domain is based on an annular packing consisting of a curved β-sheet (21 β-strands) forming the inner surface of the solenoid (“cap”) and α-helices (18) outward from the annular β-sheet. The N-terminal’s 18 β-strands out of the total 21 correspond to 18 LR repeats; the C-terminal’s three (or two in the case of StTIR1c) β-strands form the C-terminal cap, which includes the adenylate cyclase (AC) motif (localized mainly in the second β-strand). The LRR domain contains auxin- and IP6-binding sites and the interface for interaction with Aux/IAA proteins, while the F-box region mediates the interaction with SKP1.

The electrostatic potential of TIR orthologs has characteristic features (Figure 8e). On the surface of the cap/solenoid adjacent to the F-box, there is a large positively charged region, formed mainly by the side chains of arginines and lysines (sometimes also histidines), which serves as an IP6-binding site. On the opposite side of the ring, a negative charge predominates, with the exception of an auxin-binding site, which includes a region complementary to the carboxyl group of auxins. Calculations made in the Flare designer software (version 6.1.0) showed the electrostatic complementarity of auxin molecules to all potato receptors, comparable to the TIR1 Arabidopsis receptor. The 2,4-D ligand was distinguished by reduced complementarity, in agreement with experimental data [33].

IAA is bound to the active site through hydrogen bonds between the carboxyl group of the ligand and the side chains of Arg554 and Ser589 (numbering by canonical receptor alignment, Figure S1 and Figure 8b,d) and hydrogen bonding of the NH-group of the auxin heterocycle to the oxygen of the Leu590 backbone, as well as through a series of hydrophobic interactions with other amino acids. 1-NAA lacks a hydrogen bond with Leu590 due to the absence of an NH group in the aromatic part of the ligand. 2,4-D forms a direct hydrogen bond only with Arg554. Key non-mediated hydrogen bonds with ligands, similar to those in the reference TIR1, are retained in all five potato receptors, with the exception of StAFB4, in which serine at position 544 is replaced by alanine.

Thus, the homology models of presumable canonical auxin receptors of DM potatoes showed structures quite similar to the Arabidopsis TIR1 template and capable of specifically binding a hormone (auxin) and a cofactor (phosphoinositol) and interacting with proteins, including participation in SCF complex formation (Figure 8c). Along with the expression data, this leaves no doubts about full functionality of potato TIR1 orthologs in planta.

#### 2.4.2. Modeling of 3D Structure of ABP1 Orthologs—Extranuclear Auxin Receptor

**Figure 9:**
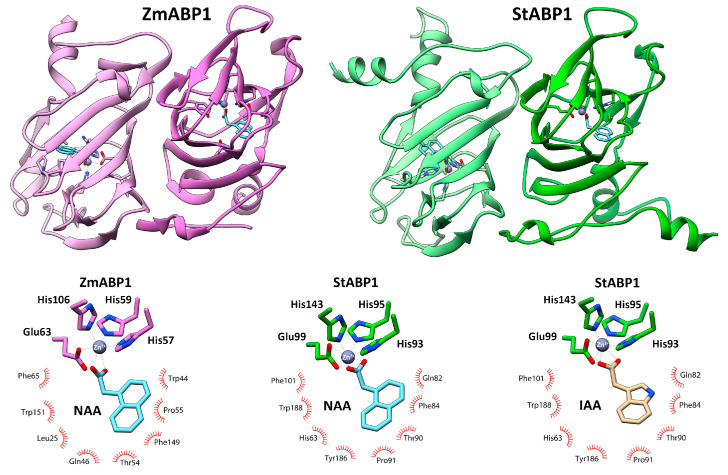
The 3D structure of the auxin-binding proteins of the ABP1 family. Comparison of the ABP homology model of potato StABP1 (green) with the crystal structure of maize ZmABP1 (pink) (PDB ID: 1LRH) [79]. **Top**, full-length ABP dimers. **Bottom**, ligand-binding patterns: NAA to ZmABP1 (**left**) in the crystal structure; NAA to StABP1 (**center**) in the homology model; and docking of IAA to the StABP1 model (**right**). Red arcs indicate residues that form hydrophobic contacts with the ligand, according to the LigPlot analysis. The blue sphere is the Zn^2+^ ion.

A model of the dimer of the StABP1 protein was generated in a complex with NAA, based on the template crystal structure of ZmABP1 from maize (PDB ID: 1LRH) [79]. The resulting model showed that StABP1 is structurally and functionally a genuine ortholog of ZmABP1 (Figure 9). The structure of these proteins is a β-jellyroll “barrel” formed by two antiparallel β-sheets, one of which includes five β-strands, while the second includes six. There is an unfolded region at the N-terminus from the “barrel”, starting (in the case of the StABP1 model) with a small α-helix; a short helix is also present at the C-terminus. The ligand-binding site is localized in the “barrel” lumen, between two β-sheets. The mode of auxin binding in ABP1 differs significantly from that of the TIR1 superfamily. In ABP1, the interaction involves the Zn^2+^ ion, which is held by four charged residues (three histidines and one glutamate) and forms bonds with the carboxyl group of auxin. In this case, the aromatic part of auxin is retained due to a number of hydrophobic interactions.

In addition to the StABP1 model with bound NAA, a complex of StABP1 with IAA was also generated by molecular docking. Thus, two structures were compared with the reference ZmABP1 (Figure 9). The zinc ion was coordinated in StABP1 by residues similar to ZmABP1, namely His93, His95, His143, and Glu99. The ligand-binding pockets of the compared maize and potato proteins differed in composition of the residues that form hydrophobic contacts with the ligand. In the case of StABP1, the main ones are His63, Gln82, Phe84, Thr90, Pro91, Phe101, Tyr186, and Trp188. As for the binding of IAA to StABP1, according to the docking results, it has no significant structural differences from the interaction of NAA with StABP1. Hence, structure modeling of the potato ABP1 ortholog clearly demonstrated the ability of StABP1 protein to specifically bind auxin—an important trait of every functional auxin receptor.

#### 2.4.3. Modeling of Other Putative Components of Auxin Signaling in Potato

**Figure 10:**
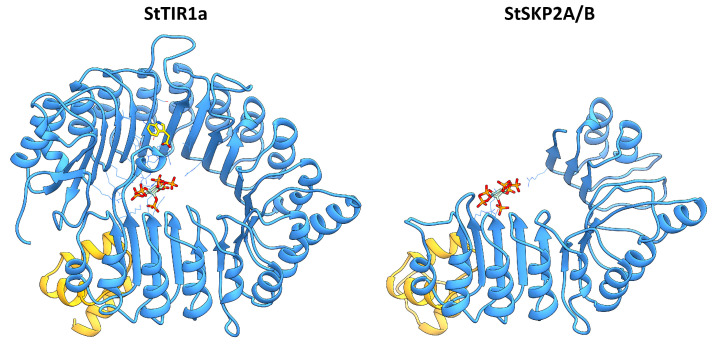
Comparison of models of the canonical receptor StTIR1a with a potential protein involved in noncanonical signaling, StSKP2A/B. Noteworthy is the absence of the LRR domain fragment (blue) in StSKP2A/B, which is involved in auxin binding. The colors of the ligand atoms are similar to those in Figure 8. The F-box domain is marked yellow.

Another protein involved in noncanonical auxin signaling in Arabidopsis is SKP2A. We modeled AtSKP2A and its closest, albeit indirect, ortholog StSKP2A/B, which turned out to be spatially similar to canonical auxin receptors, except for the absence of the C-terminal half of the LRR domain (Figure 10). Clearly, StSKP2A/B cannot bind auxin similarly to canonical TIR receptors, because of the absence of that half of the LRR domain horseshoe that is directly involved in ligand binding. Moreover, full-fledged binding of IP6 also seems unlikely, due to the absence of an essential part of the positively charged residues forming the binding site. However, we took into account the previously published data on auxin binding by AtSKP2A protein based on experiments [61]. Considering these data, we docked IAA to the putative ligand-binding site at StSKP2A/B. The results show partial similarities with the docking results in AtSKP2A and in general confirmed the ability to bind auxin, although with less complementarity (and hence less affinity) between the ligand and the binding site than in the case of canonical receptors. Focusing on the auxin-binding site that was established for Arabidopsis SKP2 [61], there is only one residue that distinguishes AtSKP2A from AtSKP2B: Leu128, replaced by Ser in the B isoform, whereas in the case of StSKP2A/B, Phe is in this position. Thus, StSKP2A/B differs from both A and B isoforms of AtSKP2 in this aspect, although it is closer to AtSKP2A, at least regarding the hydrophobicity property of this variable residue.

Next, we attempted to assess the ability of ARF3/ETT orthologs to perform the functions of auxin receptors. Molecular modeling and structure disorder prediction confirmed that StARF3 and AtARF3 lack the PB1 domain; moreover, the entire C-terminal part is markedly disordered (Figure S7), despite the fact that some authors note the presence and special role in ARF3/ETT of the so-called ES-specific domain downstream of the DBD domain [46]. Therefore, we have searched for potential auxin-binding sites in both domains. Although two potential auxin-binding pockets in DBD domain of the ETT protein have been detected (Figure S8), there is no enough evidence that they are relevant.

**Figure 11:**
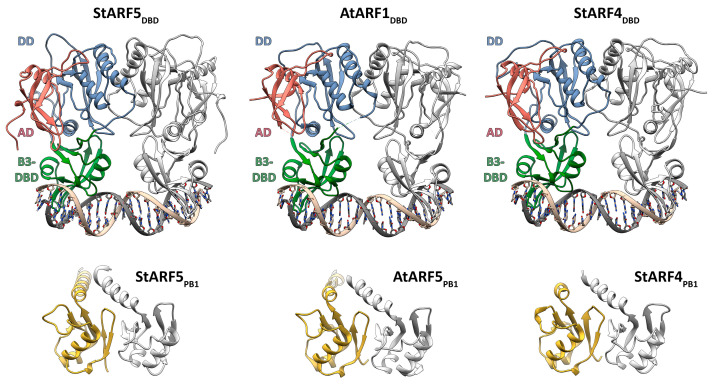
Spatial structure of canonical ARF auxin signaling transcription factors. **Top**—structures of the dimer DBD domains in complex with the promoter-like DNA sequence; DBD subdomains are highlighted in color: B3 DBD is green, DD is blue, and AD is red. **Bottom** shows dimers of PB1 (III/IV) domains. Potato StARF4 (**right**) and StARF5 (**left**) protein homology models are compared with the crystal structures (**center**) of the DBD domain of AtARF1 (PDB ID: 4LDX) [80] and the PB1 domain of AtARF5 (PDB ID: 4CHK) [81] of *A. thaliana*.

A close paralog of ARF3 in potato is StARF4; phylogenetically, both proteins form a compact ETT group (Figure 3). The question arose of whether StARF4 also belongs to the ETT group functionally, i.e., how likely is its involvement in noncanonical auxin signaling? Using the template crystal structures of the respective homologs, models of StARF4 and StARF5 proteins were constructed (Figure 11).

Models of dimers of transcription factors StARF4 and StARF5 proteins were built in a complex with a promoter-like DNA sequence (Figure 11, top). The structure of the DNA-binding domain includes eight α-helices and four β-sheets, and β-strands in each of them are oriented antiparallel to each other. The first and fourth β-sheets include five β-strands, the second three β-strands, and the third four β-strands.

The DNA-binding domain can be divided into three subdomains: B3 DBD, AD, and DD. The B3 DBD subdomain is responsible for direct interaction with DNA. The following structural elements are involved in DNA binding: the third β-sheet, loops conjugated to it, and three adjacent α-helices: α3, α4, and α5. The DD subdomain is responsible for the dimerization of the DNA-binding domain; it is formed from two regions in the aa sequence separated by the B3 DBD subdomain. The dimerization interface involves the aa of the 1st β-sheet, its conjugated loops, and α2 and α6 helices.

Models for the oligomerization PB1 (or III/IV) domain homodimers of potato StARF4 and StARF5 proteins were also built (Figure 11 bottom). The structure of this domain is represented by a central β-sheet consisting of five parallel/antiparallel β-strands and six α-helices. A feature of PB1 domain is the mode of interaction of neighboring subunits, termed “head-to-tail”, which refers to the interaction of interface 1 of subunit A with interface 2 of subunit B, localized on the opposite side of the protein surface, relative to interface 1.

Thus, StARF4 was found to be quite similar in spatial organization to the StARF5 protein, an ortholog of AtARF5/MP, the classical transcriptional regulator of the auxin-signaling system. The model showed that StARF4 possesses complete DNA (DBD)- and protein (PB1)-binding domains (Figure 11). It follows that StRF4 is more likely to be involved in canonical, rather than alternative, auxin signaling.

**Figure 12:**
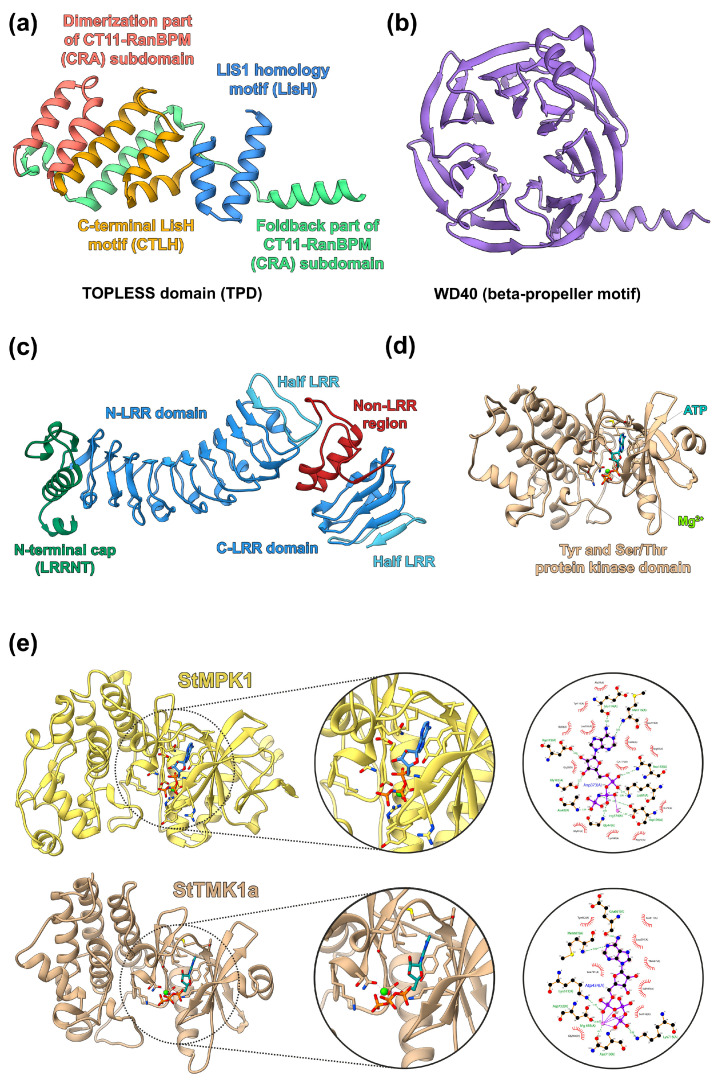
Molecular models of StTPL (**a**,**b**), StTMK1a (**c**–**e**), and StMPK1 (**e**) functional domains. (**a**) De novo model of StTPL TOPLESS domain (TPD). (**b**) De novo model of the upstream (N-terminal) WD40 domain of StTPL. (**c**) Homology model of StTMK1a LRR domains. (**d**) Homology model of StTMK1a Tyr and Ser/Thr protein kinase domain in complex with ATP molecule and Mg^2+^ ion. (**e**) Comparison of StMPK1 and StTMK1a protein kinase domains’ molecular models and their active sites. Homology model of StMPK1 protein kinase domain constructed in complex with phosphoaminophosphonic acid-adenylate ester (ANP) molecule and Mg^2+^ ion. Active sites are highlighted and magnified. The circles on the right show schemes of interactions with ligands obtained in the LigPlot program. StMPK1 stained yellow, StTMK1a sandy brown. In the ligands, oxygen atoms are colored red, nitrogen atoms are blue, phosphorus atoms are orange, carbon atoms are colored light blue in ANP and aquamarine in ATP. Mg^2+^ ions are represented as green spheres.

For better illustration of the domain structure of the proteins under studies (Figure 1), models of potato proteins of the TPL, TMK, and MPK families were built. The structure of the TOPLESS domain (TPD) of StTPL is conserved as in Arabidopsis and consists only of α-helices (Figure 12a). Five N-terminal α-helices form LISH (Lis1 homology motif) and CTLH (C-terminal LisH motif) motifs. The CTLH motif is followed by the CT11-RanBPM (CRA) subdomain, with the first two N-terminal α-helices forming the dimerization part while the remaining C-portion constitutes the foldback part of the subdomain.

At the C-terminus of the StTPL, there are two WD40 domains connected by a region including an α-helix and a short unfolded region. The structure of both WD40 domains consists of seven-bladed beta propellers (Figure 12b) and includes seven β-sheets, each consisting of four antiparallel β-strands. The β-sheets are twisted, resembling a propeller blade, in the circular structure of the WD40 domain.

The extracytosolic part of the protein kinase StTMK1a consists of two LRR domains separated by a short structure (Non-LRR region) including two α-helices (Figure 12c). These two LRR domains are unequal in size: N- and C-LRR domains include ten and three complete LR repeats, respectively. In addition, both domains terminate with an incomplete half-LRR at the C-termini. The N-terminus of N-LRR domain contains N-terminal cap (LRRNT), which consists of several α-helices and one β-strand, the latter being part of a common β-sheet with LR repeats. The cytosolic portion of StTMK1a is represented by Tyr and Ser/Thr protein kinase domains and was modeled in complex with ATP molecule and Mg^2+^ ion. This cytosolic part shares a typical structure with the protein kinase domain and can be conventionally divided into two lobes. The smaller N-terminal lobe (to the right of the ligand in Figure 12d) is formed by a β-sheet structure consisting of five antiparallel β-strands and two α-helices. The C-terminal lobe (to the left of the ligand in Figure 12d) is larger and consists predominantly of α-helices. In the cleft between these lobes, an active site, which binds ATP and Mg^2+^ ions, is located.

The model of StMPK1, though less probable as a component of the auxin signaling system in potato, was also built. Its kinase domain was modeled in a complex with a phosphoaminophosphonic acid-adenylate ester (ANP) molecule (a more stable analogue of ATP) and Mg^2+^ ion. The kinase domain of StMPK1, although sharing the generally conserved spatial organization, nevertheless differs from StTMK1a, both in the folding features and the active center (Figure 12e). In particular, in the N-terminal lobe, the central β-sheet is preserved, but one of two α-helices is degenerated, and another β-sheet consisting of three β-strands is formed in its place. In the active site, despite the retention of the main conserved residues and the similar position of the substrate, the position of the Mg^2+^ cofactor and the set of residues holding it differ between StTMK1a and StMPK1 (Figure 12e, highlighted circles).

#### 2.4.4. Main Conclusions of Molecular Modeling and Docking

The work on 3D modeling of protein structures and docking has provided a unique opportunity to visualize and clearly compare potential potato auxin receptors both among themselves and with Arabidopsis/maize orthologous proteins. Using modern modeling software and potato as an example, we were able to observe in great detail the spatial structure of the key auxin signaling proteins—presumable auxin receptors. As a result of this work, we found that in all tested parameters (general and particular structural similarity, interaction with ligands, interaction with specific partners), the putative canonical potato auxin receptors fully correspond to the canonical auxin receptor TIR1 of Arabidopsis.

The same applies to the known auxin-binding protein ABP1, whose receptor status has recently received experimental confirmation [27]. As for the other potential noncanonical receptors StARF3/ETT and StSKP2A/B, the similarity of their general and domain structures with those of Arabidopsis orthologs has been shown. This similarity is, of course, important for the fulfillment of receptor functions by these proteins, but it is not able to guarantee this fulfillment, especially as these proteins are not close orthologs of Arabidopsis counterparts. Our docking assays with auxin models failed to confirm the ability of these proteins to bind auxin with high affinity (not shown). It may be that auxin acts in this case not as a typical hormone but rather as an allosteric regulator with a relatively low affinity. Functional tests, particularly testing the ability to specifically bind to the hormone in a direct experiment, could be decisive.

## 3. Materials and Methods

### 3.1. Analysis of Auxin Signaling-Related Genes

In our work, we identified all possible genes of the currently proven components of the potato auxin signaling system. Data from the NCBI (GeneBank) database were used as the basis. The genes/proteins of doubled Phureja monoploid (DM), heterozygous diploid RH89-039-16 (RH), and autotetraploid Otava (Ot) were searched using BLASTP suite service (https://blast.ncbi.nlm.nih.gov/Blast.cgi?PAGE=Proteins, accessed on 20 February 2023), using sequences of corresponding Arabidopsis proteins. The data in Table S1 are given for SolTub_3.0 version (GCF_000226075.1). Similarly retrieved protein sequences of tomato, Arabidopsis, and rice (Japonica Group) were also used for phylogenetic analysis. The data obtained were compared with the data available in Phytozome 13 for *Solanum tuberosum* v.6.1. Information on the localization of genes on potato chromosomes was obtained from there. This database was also used to retrieve information about gene expression in various organs (http://spuddb.uga.edu/dm_v6_1_download.shtml, accessed on 20 February 2023). Only genes for which there was reason to assume their direct participation in auxin signaling, i.e., transformation of chemical signal, delivered by auxin molecule, into primary changes in cellular homeostasis, were considered. If a particular gene codes for several splice versions of transcripts, we summarized expression data from all mRNAs that encoded functional full-fledged proteins. The domain composition of protein sequences was determined using the HMMER service (https://www.ebi.ac.uk/Tools/hmmer/, accessed on 20 February 2023), the SMART service (http://smart.embl-heidelberg.de/, accessed on 20 February 2023), the Conserved Domain Database (CDD) service (https://www.ncbi.nlm.nih.gov/Structure/cdd/wrpsb.cgi, accessed on 20 February 2023), and InterPro (https://www.ebi.ac.uk/interpro/, accessed on 24 May 2023). Transmembrane domains were identified using the TMHMM-2.0 service (https://services.healthtech.dtu.dk/services/TMHMM-2.0/, accessed on 20 February 2023). In addition, protein sequence alignment was performed for a number of groups to study the functionality of individual components in more detail. Cluster analysis of gene group expression by organ was performed using Heatmapper web server (http://www.heatmapper.ca/expression/, accessed on 15 June 2023) (Figure 7 upper panel; Figures S5 and S6). The goal of this analysis was to identify major signaling participants from each protein group in each organ. Clustering method was average linkage. Distance measurement method was Pearson.

### 3.2. Phylogenetic Analysis

The evolutionary history was inferred using the neighbor-joining method [82]. The optimal tree with the minimized sum of branch length is shown. The percentages of replicate trees in which the associated taxa clustered together in the bootstrap test (1000 replicates) are shown next to the branches [83]. The tree is drawn to scale, with branch lengths in the same units as those of the evolutionary distances used to infer the phylogenetic tree. The evolutionary distances were computed using the p-distance method [84] and are in the units of the number of amino acid differences per site. The analysis involved sequences of 6 to 53 amino acids. All positions containing gaps and missing data were eliminated. Evolutionary analyses were conducted in MEGA6 [85].

### 3.3. Plant Growth Conditions

Gene expression at two stages of tuberization (stolon and tuber stages) was studied. Potato cuttings from cv. Désirée were initially grown in vitro on agarized MS medium for one month with a 16 h photoperiod (long day, LD) and at 20–22 °C. The resulting young plants were then transferred to an aeroponics unit. There, the plants were initially grown for another month under LD conditions, with 24 °C at leaf area and 19 °C at root area (dark chamber). Roots were sprayed by nutrient solution as described in Çalışkan et al. [86] every 15 min for 30 s. The photon flux at the leaf level was 100 μmol m^−2^ s^−1^, provided by fluorescent lamps (T8 Philips TL-D 36W/33-640 G13, 4100 K). During this time, potato plants adapted to the new growing conditions and attained the desired biomass. Then, the cultivation was switched to short-day (SD) under conditions of 10 h light and 14 h dark at the same temperature and light intensity. One month later, plant organs—leaves, stems, roots, stolons, and tubers—were collected separately and frozen in liquid nitrogen for gene expression analysis.

### 3.4. Quantitative Real-Time PCR

Total RNA was extracted from 100–250 mg of fresh tissues by means of the RNeasy Plant Mini Kit (Qiagen, Hilden, Germany) and treated with RNAse-free DNase I (1 U/μL) (Evrogen, Moscow, Russia). cDNA was synthesized on the RNA template with MMLV reverse transcriptase (Evrogen) according to manufacturer’s protocol. The absence of genomic DNA in cDNA samples was confirmed by PCR with primers of intron-containing fragment of patatin gene. Quantitative gene expression was determined by quantitative real-time PCR (RT-qPCR) (Figure 7, lower panel) using Light-Cycler 96 (Roche, Basel, Switzerland). DNA was amplified with qPCRmix-HS SYBR (Evrogen); primers are listed in Table S3. Gene-specific primers were designed by means of the Primer-Blast program (https://www.ncbi.nlm.nih.gov/tools/primer-blast/, accessed on 28 July 2021), ensuring primer uniqueness; the best primer pairs were selected using the OligoAnalyzer tool (https://eu.idtdna.com/, accessed on 28 July 2021). When possible, primers crossing the exon/intron boundary were chosen. Primer quality was additionally validated by melting amplicons generated by RT-qPCR as well as by amplicon size and purity determination using electrophoresis in 1% agarose gel. The conditions for RT-qPCR were as follows: pre-denaturation at 95 °C for 60 s, followed by 35 cycles of denaturation at 95 °C for 30 s, annealing at 60 °C for 15 s, and extension at 72 °C for 15 s. DNA sequences encoding potato elongation factor EF1a (GenBank acc. No. AF126551) and cyclophilin CYC (GenBank acc. No. AF126551) were employed (with similar results) as reference genes [87]. Each value of transcript content used to calculate average values in Figure 7 (lower panel) represents the mean of three technical replicates.

### 3.5. Statistics

The biological experiment was repeated three times, and the underground and aboveground organs were analyzed in four and two biological replicates, respectively. In real-time PCR, there were three analytical replicates for each sample, and the mean values and standard deviations were calculated using the Excel program. More than twofold differences in gene expression values were considered significant. To evaluate more quantitatively a similarity degree between expression patterns in potato ecotypes, pairwise correlation Spearman coefficients have been determined using Excel- and SigmaPlot-provided algorithms.

### 3.6. Molecular Modeling

Molecular modeling was performed using the YASARA Structure software (version 22.9.24) [88]. A slow modeling protocol was chosen. The number of PSI-BLAST iterations was set to 3, and the E-value of PSI-BLAST was set to 0.1. The number of alignments per template was set to 5. The terminal extension was set to 10, and the number of loop samples was set to 50. The YASARA modeling protocol consists of a number of model optimization steps, including energy minimization, side-chain rotamer fine-tuning, loop optimization, hydrogen-bonding network optimization, and finally, creating a hybrid model in which imperfect regions in the top-scoring model are iteratively replaced with corresponding fragments from the other models. Both single-template and multi-template algorithms were used in the modeling, in different cases. The structures that served as templates are listed below.

Crystal structures of the Arabidopsis AtTIR1 receptor complex with IP6, the AtSKP1a protein, the peptide fragment of the AtAux/IAA7 protein, and three different ligands—IAA (PDB ID: 2P1Q), NAA (PDB ID: 2P1O) and 2,4-D (PDB ID: 2P1N) [66]—were used to model the potato family TIR1 proteins. To model the StABP1 protein, the crystal structure of *Zea mays* ABP1 in complex with NAA and Zn^2+^ (PDB ID: 1LRH) was used as a template [79].

Models of the DNA-binding domain (DBD) dimers of potato ARF proteins were constructed using the template crystal structure of the ARF1 DNA-binding domain of *A. thaliana* (PDB ID: 4LDX) [80]. Another crystal structure of AtARF1 (PDB ID: 6YCQ) [89] and the structure of MpARF2 of *Marchantia polymorpha* (PDB ID: 6SDG) [90] served as additional templates. The crystal structure of the homodimer of the ARF5 oligomerization domain of *A. thaliana* (PDB ID: 4CHK) was used as a template to model the PB1 domains of potato ARF proteins [81].

StARF3 and AtARF3 models were constructed by both de novo and homology methods. The AtSKP2A, AtSKP2B, and StSKP2A/B proteins were modeled using the Arabidopsis AtTIR1 receptor crystal structure (PDB ID: 2P1P) [66] and the crystal structure of *Homo sapiens* GGTase3-FBXL2-SKP1 complex (PDB ID: 6O60) [91] as templates. The structure of full-sized StTPL was built by de novo modeling and analyzed, using results of the preceding works [73,92], including the crystal structure of *A. thaliana* TOPLESS N-terminal domain (PDB ID’s: 5NQS, 5NQV). The extracytosolic portion of StTMK1a was constructed by homology modeling using the structure of *A. thaliana* AtTMK1 (PDB ID: 4HQ1) as a template [75]. The cytosolic portion of StTMK1a is represented by the Tyr and Ser/Thr protein kinase domain and was modeled in complex with ATP molecule and Mg^2+^ ion using structure of the *A. thaliana* FER Receptor Kinase (PDB ID: 7XDV) [93] as the template. The StMPK1 protein kinase domain model was constructed in complex with phosphoaminophosphonic acid-adenylate ester (ANP) molecule and Mg^2+^ ion, using the template structure of the *A. thaliana* Mitogen-Activated Protein Kinase 4 (MPK4) (PDB ID: 7W5C) [94].

Additional modeling was accomplished in Modeller (version 9.20) [95] using an automodel class for comparative modeling. For each protein, 200 models were built, and the best model was selected according to the value of DOPE (discrete optimized protein energy) score [96] calculated by Modeller. De novo modeling was performed using AlphaFold [97], AlphaFoldmultimer [98], OmegaFold [99], and IntFold [100] services. The first three are implemented in the COSMIC^2^ service (https://cosmic-cryoem.org/, accessed on 27 June 2023) [101]. IntFold (https://www.reading.ac.uk/bioinf/IntFOLD/, accessed on 4 February 2023) has also been used for structure disorder prediction.

Docking was performed using VINA [102] with default parameters. The setup was performed with the YASARA molecular modeling program [88]. Minimum of ligand RMSD was set to 5.0 Å. The ligand pose with the best energy score was selected for further use. Structure data file (Model SDF) of 1H-indol-3-ylacetic acid from PDB was used for docking (PDB ID: IAC). The models were further refined by short molecular dynamics using the md_refine macro in YASARA Structure software (version 22.9.24) [103]. Temperature was set to 298 K, density was set to 0.997, and pH was set to 7.2.

Calculation of electrostatic complementarity for protein–ligand interactions was performed using the Cresset Flare Designer software (version 6.1.0) [104]. Proteins were prepared using the Normal calculation method, intelligent capping of chains, and auto-extract of ligand. Active site size was set to 6 Å. Water molecules were removed outside the active site. For nuclear proteins, the pH value 7.2 was set for calculations. Small side chain movements were allowed. Atoms from residues with incomplete backbone were removed. A search for potential binding sites was performed using the PrankWeb web service (https://prankweb.cz/, accessed on 8 March 2023) [105].

Analysis of protein–ligand interactions was performed in LigPlot (version 2.2.8) with default parameters [106]. For hydrogen bond calculation, maximum H-A distance was 2.7 Å and maximum D-A distance was 3.35 Å. For non-bonded contacts, minimum contact distance was 2.9 Å and maximum contact distance was 3.9 Å. Calculations were performed for “hydrophobic-any” contacts (instead of “hydrophobic-hydrophobic” or “any”) where hydrophobic atoms were C or S. Treatment of CONECT records was used if sensible.

Visualization of spatial structures and coloring of the protein surface by electrostatic potential was carried out in UCSF Chimera (version 1.14) [107]. Electrostatic potentials in Chimera were calculated using Coloumbic Surface Coloring, ranging from −10 to 10 kcal/(mol*e), with dielectric constant set to 4.0 and distance from surface equal to 1.4. Molecular dynamics simulations were performed with YASARA Structure software (version 22.9.24) [88].

## 4. Conclusions

Auxins are highly important classical phytohormones, and they determine and mirror the basic properties of the plant hormonal system. Despite the common traits of plant and animal hormonal regulation (low concentrations of hormones, their movement from the synthesis site to the targeted one, the crucial role of the receptors in hormone sensing, etc.), there is a fundamental difference between these two systems. The animal system consists of a large variety of hormone types (more than 100) and is organized in a rather linear way: each hormone has a distinct synthesis site and normally acts through a single or, less frequently, through a pair of similar receptors [108]. In contrast, the plant system includes far fewer hormone types (about 10–12) but has multiple sites for each hormone biosynthesis and perception, often involving the same tissue (paracrine action).

As a result, phytohormones such as auxin and cytokinins form in plant defined concentration gradients that are fairly stable in time and space. Such specific hormonal patterns control cell division and differentiation, which underlie organ emergence and growth. Long ago, taking into account this divergence between plants and animals, it was postulated that “The presence of several receptors acting at different levels for the same phytohormone may partially compensate for the general ‘ormonal deficiency’ of plants in comparison with animals, in which one type of acceptor is characteristic of each given hormone. Different types of receptors can apparently “coexist” in the same (plant) cells…” [109,110]. According to the current knowledge of auxin signaling, this prediction turned out to be correct. In Arabidopsis, several receptor proteins that perceive the auxin signal at different subcellular sites were found [28,36,37,52]. Our analysis of the potato genomes evidenced the principal auxin perception apparatus in different potato ecotypes, despite the possible substitution of some particular genes. All studied potato cultivars, regardless of their ploidy, retain a set of genes encoding compounds of the main canonical pathway for auxin signaling, which consists of receptors (orthologs of TIR1/AFB proteins), ARF-homologous transcription factors, and Aux/IAA-homologous repressors. Although the total number of these genes per cell differs depending on genome ploidy (40 genes in doubled monoploid Phureja versus 94 genes in autotetraploid Otava; together with *TPL/TPRs*, 46 and 108 genes, respectively), these canonical gene sets should be considered qualitatively identical because they perfectly match to each other. Apart from this, all studied potato ecotypes possess genes presumably encoding noncanonical/alternative auxin signaling (orthologs of *ABP1*, *ARF3*-, and *SKP2A*, as well as protein kinase-encoding *TMK1/4* and *MPK* genes (totalling about 16 Phureja versus 40 Otava genes)).

It is worth noting that the canonical auxin signaling seems to not be limited by targeted repressor degradation in proteasomes. A recent study has shown an adenylate cyclase activity at the C-terminus of the TIR1/AFB canonical auxin receptors [68] (Figure 1). The generated cAMP was found to be important for auxin-mediated regulation of nuclear gene expression.

Transcriptomic studies confirmed the similarity between different potato ecotypes in their pattern of auxin signaling gene expression. Regardless of their ploidy, all potatoes preferably use orthologs of *StARF1*, *StARF2a*, *StARF19a, StARF6a*, *StARF6b*, *StARF8b*, and *StARF5* transcription factor (*TF*) genes, as well as *StIAA1, StIAA3*, *StIAA23*, *StIAA8* and *StIAA2* genes encoding TF repressors, obviously for canonical signaling. This general similarity of the expression patterns was corroborated by statistical analysis. However, detailed analysis also revealed marked differences between these ecotypes in the expression of particular auxin signaling-related genes, especially in the roots. In DM potatoes, *StARF19a* dominates as a transcription activator, while in RH, *StARF5* appears to play the same role instead. In contrast, in Désirée roots, both foregoing *TF* genes were active. Close orthologs of the genes for noncanonical auxin signaling (*ABP1*, *TMK1/4*, *ARF3/ETT*, etc.) in the studied potato genomes were also effectively expressed. Thus, potatoes, like Arabidopsis, obviously use various pathways for auxin signaling, though some important details of these pathways in these species can be different.

Results of our study lend support to the functioning in potato of at least two alternative pathways triggered by noncanonical auxin receptors StABP1 and also probably StARF3/ETT. These pathways seem to exist in different potato cultivars regardless of their genotype/ploidy (DM, RH, or tetraploid). In contrast, the noncanonical pathways interfering with the core signaling and based on Degron-free Aux/IAA repressors seem much less probable in potatoes than in Arabidopsis. The latter has at least seven such “defective” genes, whereas DM potato has only two (*StIAA17* and *32*). The Arabidopsis noncanonical genes were shown to be expressed in planta (http://travadb.org/browse/, accessed on 20 February 2023), while both their orthologs in potato seemingly were not (Figure 7). This may indicate that these two genes have become pseudogenes in DM potato. The complete deletion of the *IAA17* gene from the Otava genome supports this hypothesis. At the same time, the presence of additional noncanonical auxin signaling pathways in potato distinct from those in Arabidopsis cannot be ruled out. In any case, auxin signaling in plants including potato should no longer be considered a linear process of signal transduction or even signaling cascade but rather a complex signaling network involving several simultaneous processes (canonical and noncanonical) of multidirectional signal transmission. This study can serve as a basis for targeted modifications of the auxin signaling system in potatoes.

## Data Availability

Not applicable.

## Supplementary Materials

The supporting information can be downloaded at https://www.mdpi.com/article/10.3390/ijms241411408/s1.
