# Supplementary material for: Canonical and Alternative Auxin Signaling Systems in Mono-, Di-, and Tetraploid Potatoes
Source: Int J Mol Sci. 2023 Jul 13;24(14):11408. doi: 10.3390/ijms241411408 (PMC10380454; doi:10.3390/ijms241411408)
Supplement: Supplementary file 1 [file ijms-24-11408-s001.zip › ijms-2385745-supplementary.pdf]

Supplementary data

to article by Lomin et al. “Canonic and Alternative Auxin Signaling... ”

Table S1 (beginning). Potato genes/proteins presumably involved in auxin signaling, exemplified by DM 'Phureja' and tetraploid 'Otava' cultivars

Gene name	Gene symbol DM	Protein DM	Amino acids	Gene symbol Otava	Protein Otava	Amino acids
**TIR1/AFB related**	5			13		
StTIR1a	LOC102578999*	XP_006352806.1	580	KY289_028764*	KAH0660016.1	580
				KY290_029572*	KAH0750340.1	580
StTIR1b	LOC102583018*	XP_006359432.1	581	KY285_036735*	KAH0640149.1	581
				KY284_037768*	KAH0634982.1	581
				KY290_037414*	KAH0738709.1	581
StTIR1c	LOC102578728*	XP_006362983.1/84.1	605/*510*	KY289_029125*	KAH0660377.1	605
				KY285_025893*	KAH0664687.1	604
				KY284_026005*	KAH0661074.1	604
StAFB4	LOC102578575*	XP_006338135.1	617	KY289_021518*	KAH0683766.1	622
				KY285_021251*	KAH0694154.1	623
				KY284_021302*	KAH0680217.1	613
StAFB6	LOC102595865*	XP_015164173.1/ XP_006347140.1	589	KY285_014045*	KAH0718014.1	589
				KY289_014659*	KAH0697177.1	589
**ARF activators**	9			20		
StARF5	LOC102590158*	XP_006342026.1	929	KY284_021699*	KAH0680614.1	929
StARF6a	LOC102606235*	XP_006357892.1/93.1	893/892	KY289_030941*	*KAH0653263.1*	894
				KY284_030624*	*KAH0650712.1*	892
				KY285_032006*	*KAH0657124.1*	892
StARF6b	LOC102598519*	XP_006351989.1/90.1	884/883	KY289_011869*	*KAH0706793.1*	884
				KY290_011314*	KAH0774177.1	882
				KY284_011391*	*KAH0709964.1*	882
StARF8a	LOC102597704*	XP_006343312.1/*13.1/ XP_015162339.1*	848/*752/752*	KY284_018481*	KAH0687928.1	842
StARF8b	LOC102598513*	XP_006350451.1/52.1/ XP_015165582.1	840/839/*743*	KY284_015156*	KAH0700941.1	839
				KY290_015847*	KAH0771866.1	841
StARF8c	LOC102597963*	XP_006350449.1/50.1	838/837	KY289_013757*	KAH0696275.1	830
				KY284_015157*	KAH0700942.1	996
				KY289_015516*	KAH0698034.1	796
StARF19a	LOC102594021*	XP_006353789.1	1157	KY284_030511*	KAH0650599.1	1167
				KY289_030846*	KAH0653168.1	1119
StARF19b	LOC102592438*	XP_006365636.1	1114	KY289_030181*	KAH0652503.1	1112
				KY284_029972*	KAH0650060 .1	1113
				KY285_030018*	KAH0655136.1	1110
StARF19c	LOC102587769*	XP_006360656.1/57.1	1097	KY284_024163*	KAH0673076.1.1	1095
				KY289_024436*	KAH0669943.1	1096

* and ** means that the gene presumably belongs to canonical or alternative auxin signaling system, respectively.

Table S1 (continuation 1). Potato genes/proteins presumably involved in auxin signaling, exemplified by DM 'Phureja' and tetraploid 'Otava' cultivars

Gene name	Gene symbol DM	Protein DM	Amino acids	Gene symbol Otava	Protein Otava	Amino acids
**ARF inhibitors**	7(6)			12(11)		
StARF1	LOC102604408*	XP_015165011.1/ XP_006349280.1/81.1/82.1	654	KY289_003002*	KAH0731814.1	679
				KY290_002801*	KAH0783203.1	679
				KY284_002912*	KAH0727047.1	679
StARF2a	LOC102582348*	XP_006341482.1	845	KY284_018915*	KAH0688362.1	849
StARF2b	LOC102594206*	XP_006346635.1	829	KY284_012253*	KAH0710826.1	829
				KY289_010799*	KAH0705723.1	829
StARF13	LOC102587577	*XP_015163537.1/38.1/39.1*	*650/645/573*	KY285_025646	*KAH0677845.1*	*657*
StARF18a	LOC102586095*	XP_006339723.1/24.1/25.1	708/705/695	KY289_002568*	KAH0731380.1	687
				KY284_002456*	KAH0726591.1	687
StARF18b	LOC102605272*	XP_006343828.1	655	KY289_012319*	KAH0707243.1	650
StARF18c	LOC102582092*	XP_006352240.1/41.1/ *XP_015166335.1*	685/683/*586*	KY284_034399*	KAH0646515.1	685
				KY289_032705*	KAH0641731.1	685
**ETTs**	2(0)			7(1)		
StARF3	LOC102582172**	*XP_006364312.1/13.1/15.1*	*748/748/747*	KY284_013906**	*KAH0699691.1*	*747*
				KY289_014561**	*KAH0697079.1*	*793*
				KY290_014506**	*KAH0770525.1*	*883*
				KY285_015336**	*KAH0719305.1*	*747*
StARF4	LOC102602099*	*XP_006340145.1*	*811*	KY289_009905*	KAH0713946 .1	811
				KY284_008438*	KAH0715533.1	811
				KY285_009231*	KAH0747574.1	811
**Abnormal ARFs**	6(0)			17(0)		
StARF10a	LOC102586871	*XP_006340187.1/88.1/ XP_015170635.1/37.1*	*699/699/696/696*	KY290_008530	*KAH0777119.1*	*699*
				KY284_008460	*KAH0715555.1*	*699*
StARF10b	LOC102599257	*XP_006365732.1*	*676*	KY284_028218	*KAH0663287.1*	*676*
				KY285_029414	*KAH0668208.1*	*676*
				KY289_029555	*KAH0660807.1*	*676*
StARF10c	LOC102585790	*XP_006363667.1*	*648*	KY289_006066	*KAH0723022.1*	*641*
				KY284_005740	*KAH0720710.1*	*642*
StARF11	LOC102584504	*XP_006367077.2*	*190*	KY284_017614	*KAH0687061.1*	*197*
				KY285_017608	*KAH0703330.1*	*151*
				KY290_018647	*KAH0762574.1*	*194*
				KY289_017768	*KAH0690410.1*	*198*
StARF16	LOC102592252	*XP_006354327.1*	*696*	KY289_017768	*KAH0635551.1*	*694*
				KY290_035963	*KAH0737258.1*	*694*
				KY285_037550	*KAH0640964.1*	*694*
StARF17	LOC102588218	*XP_006364958.1*	*602*	KY284_009116	*KAH0716211.1*	*602*
				KY285_009060	*KAH0747403.1*	*602*
				KY290_009178	*KAH0777767.1*	*591*

* and ** means that the gene presumably belongs to canonical or alternative auxin signaling system, respectively.

Table S1 (continuation 2). Potato genes/proteins presumably involved in auxin signaling, exemplified by DM 'Phureja' and tetraploid 'Otava' cultivars

Gene name	Gene symbol DM	Protein DM	Amino acids	Gene symbol Otava	Protein Otava	Amino acids
**Canonical Aux/IAAs I**	16			39		
StIAA1	LOC102577658*	NP_001275473.1	349	KY289_021579*	KAH0683827.1	384
				KY284_021361*	KAH0680276.1	349
				KY285_022426*	KAH0695329.1	400
StIAA2	102586718/IAA2*	NP_001275031.1	213	KY285_027087*	KAH0665881.1	213
				KY290_028122*	KAH0748890.1	213
				KY284_027103*	KAH0662172.1	213
StIAA3	102577631/IAA3*	NP_001275398.1	249	KY284_004248*	KAH0728383.1	499
				KY289_004459*	KAH0733271.1	499
StIAA4	LOC102596255*	XP_006356050.2	213	KY284_015710*	KAH0685157	189
				KY285_015711*	KAH0701433	189
				KY290_016407*	KAH0760334	189
StIAA6	LOC102599665*	XP_006343104.1	195	KY289_019200*	KAH0691842	193
StIAA7	LOC102599338*	XP_006343103.2	212	KY284_018141*	KAH0687588.1	212
				KY289_018299*	KAH0690941.1	216
StIAA8	LOC102595402*	XP_006343091.1	278	KY290_019205*	KAH0763132.1	262
				KY284_018152*	KAH0687599.1	266
				KY289_018306*	KAH0690948.1	366
				KY285_018168*	KAH0703890.1	347
StIAA10	LOC102587812*	XP_006366663.1	214	KY289_029132*	KAH0660384.1	214
				KY284_026025*	KAH0661094.1	214
				KY285_025922*	KAH0664716.1	214
StIAA11	LOC102587488*	XP_006366662.1	190	KY284_026026*	KAH0661095.1	190
				KY285_025923*	KAH0664717.1	190
StIAA12	LOC102586390*	XP_006350661.1	190	KY285_027088*	KAH0665882.1	190
				KY289_027289*	KAH0658541.1	190
				KY290_028123*	KAH0748891.1	190
StIAA14	LOC102583557*	XP_006356170.1	176	KY290_028702*	KAH0749470.1	135
				KY284_027702*	KAH0662771.1	136
				KY285_027711*	KAH0666505.1	139
StIAA15	LOC102590668*	XP_006352755.1	153	KY284_028530*	KAH0663599.1	159
StIAA21	LOC102578779*	XP_006349740.1	183	KY284_036577*	KAH0633791.1	182
StIAA22	LOC102592864*	XP_006362194.1	195	KY289_038044*	KAH0638129.1	195
				KY285_036949*	KAH0640363.1	195
StIAA23	LOC102593208*	XP_006362195.1	238	KY284_036993*	KAH0634207.1	239
				KY285_036950*	KAH0640364.1	236
StIAA25	LOC102577929*	XP_006353472.1	297	KY285_011423*	KAH0735716.1	295
				KY284_011464*	KAH0710037.1	373
				KY290_011378*	KAH0774241.1	295
				KY289_012904*	KAH0707828.1	295

* and ** means that the gene presumably belongs to canonical or alternative auxin signaling system, respectively.

Table S1 (continuation 3). Potato genes/proteins presumably involved in auxin signaling, exemplified by DM 'Phureja' and tetraploid 'Otava' cultivars

Gene name	Gene symbol DM	Protein DM	Amino acids	Gene symbol Otava	Protein Otava	Amino acids
**Abnormal IAA28-like**	1			3		
StIAA9	LOC102585218	XP_015162257.1	283	KY290_019252	KAH0763179.1	283
				KY284_018202	KAH0687649.1	283
				KY289_018356	KAH0690998.1	283
**Canonical Aux/IAAs II**	3			8		
StIAA20	LOC102585970*	XP_006355888.1	322	KY285_036436*	KAH0639850.1	323
				KY284_036466*	KAH0633680.1	323
StIAA24	LOC102581585*	XP_006340886.1/ XP_015160844.1	283/282	KY290_037732*	KAH073902.1	282
				KY284_037108*	KAH0634322.1	322
				KY285_037848*	KAH0641262.1	282
StIAA26	LOC102591186*	XP_015162035.1/ XP_006342408.1	232/231	KY289_012606*	KAH0707530.1	363
				KY285_012080*	KAH0736373.1	363
				KY284_009720*	KAH0708293.1	363
**NC IAA32-like**	1			3		
StIAA32	LOC102582938**	XP_006344317.1	192	KY284_022967**	KAH0671880.1	191
				KY285_025320**	KAH0677519.1	191
				KY289_023159**	KAH0668666.1	191
**Abnormal IAA29-like**	3			3		
StIAA13	LOC102585622	XP_006353683.1	237			
StIAA16	LOC102583661	XP_006358296.1	203	KY289_032336	KAH0654658.1	203
				KY284_029800	KAH0649888.1	203
StIAA19	LOC102602017**	XP_006343525.1	219	KY289_034747	KAH0643773.1	79
**NC IAA33-like**						
StIAA17	LOC102602445**	XP_006341262.1	143			
**TPL-related**	6			14		
StTPL	LOC102581018*	XP_015161654.1/55.1/56.1	1132/1132/1130	KY284_018894*	KAH0688341.1	1130
StTPR4a	LOC102602466*	XP_006364222.1/ XP_015159310.1	1132/1130	KY290_019978*	KAH0763905.1	1132
				KY284_018884*	KAH0688331.1	1179
StTPR4b	LOC102582783*	XP_006358293.1/94.1	1134/1133	KY289_031993*	KAH0654315.1	1133
				KY285_029831*	KAH0654949	1134
				KY284_029802*	KAH0649890.1	1134
StTRP2	LOC102596120*	XP_006352935.1/36.1	1136	KY284_034652*	KAH0646768.1	1135
				KY290_035374*	KAH0742331.1	1135
				KY289_033825*	KAH0642851.1	1116
StTPR3	LOC102590171*	XP_006344707.1/08.1/09.9	1155/1132/1131	KY285_004104*	KAH0768233.1	1142
				KY290_002655*	KAH0783057.1	1143
				KY289_002850*	KAH0731662.1	1143
StTPR5	LOC102596956*	XP_006343510.1/11.1/ XP_015162492.1/93.1	1180/1142/ 1142/1066	KY289_025981*	KAH0671488.1	1180
				KY284_032787*	KAH0644903.1	1180

* and ** means that the gene presumably belongs to canonical or alternative auxin signaling system, respectively. StIAA17 and StIAA32 may be pseudogene.

Table S1 (ending). Potato genes/proteins presumably involved in auxin signaling, exemplified by DM 'Phureja' and tetraploid 'Otava' cultivars

Gene name	Gene symbol DM	Protein DM	Amino acids	Gene symbol Otava	Protein Otava	Amino acids
**SKP2A/B**	1			2		
StSKP2A/B	LOC102579014**	XP_015168274.1	365	KY289_021131**	KAH0683379.1	365
				KY284_020964**	KAH0679879.1	365
**ABP1**	1			4		
StABP1	LOC102602619**	XP_006352791.1	202	KY284_029397**	KAH0664466.1	227
				KY289_029621**	KAH0660873.1	207
				KY285_028614**	KAH0667408.1	164
				KY290_029562**	KAH0750330.1	164
**TMK1 related**	9(8)			23		
StTMK1a	LOC102605763**	XP_006350960.1	938	KY289_007645**	KAH0711686.1	938
				KY284_007373**	KAH0714468.1	938
StTMK1b	LOC102589646**	XP_006346490.1	942	KY289_004663**	KAH0733475.1	942
StTMK1c	LOC102589646	*XP_015163711.1*	468	KY289_004663**	KAH0733475.1	942
StTMK4a	LOC102599434**	XP_006360918.1	932	KY289_037438**	KAH0637523.1	932
				KY289_004663**	KAH0733475.1	942
				KY284_037238**	KAH0634452.1	932
StTMK4b	LOC102578437**	XP_006349904.1	921	KY285_009583**	KAH0733876.1	921
				KY289_013042**	KAH0707966.1	921
StTMK4c	LOC102581637**	XP_006366998.2	942	KY284_036272**	KAH0633486.1	942
				KY289_037918**	KAH0638003.1	942
				KY290_036914**	KAH0738209.1	942
				KY285_036254**	KAH0639668.1	942
StTMK5a	LOC102585650**	XP_006339166.1	934	KY284_006756**	KAH0721726.1	934
				KY289_007063**	KAH0724019.1	934
StTMK5b	LOC102579753**	XP_006348472.1	963	KY290_000207**	KAH0780609.1	955
				KY284_000216**	KAH0724351.1	955
				KY285_000195**	KAH0764324.1	955
				KY289_004026**	KAH0732838.1	955
StTMK5c	LOC102605581**	XP_006361096.1	978	KY289_029533**	KAH0660785.1	978
				KY285_028177**	KAH0666971.1	977
				KY284_028131**	KAH0663200.1	978
				KY290_029130**	KAH0749898.1	938
**MPK1/2/7/14 related**	2			4(2)		
StMPK_1	LOC102601775**	XP_006341972.1	372	KY285_027891**	*KAH0666685.1*	234
				KY289_029745**	*KAH0660997.1*	264
StMPK_2	LOC102592852**	XP_006340663.1	370	KY290_015059**	KAH0771078.1	370
				KY285_014474**	KAH0718443.1	392

* and ** means that the gene presumably belongs to canonical or alternative auxin signaling system, respectively.

Table S2. Spearman correlation coefficients [R_{s}] between expression patterns of DM-RH different groups of genes, which presumably take part in auxin signaling

**Genes**	**Organs**					
	**Leaves**	**Roots**	**Stolons**	**Tubers**	**Flowers**	**Petioles**
All	0,709**	0,755**	0,662**	0,813**	0,806**	0,804**
Canonic	0,716**	0,714**	0,464**	0,766**	0,933**	0,711**
Non-canonic	0,743**	0,816**	0,885**	0,852**	0,63**	0,887**

** means highly significant correlation (*P*< 0.01)

Table S3. Primers for RT-qPCR

Locus	Gene	Name	Primer
LOC102583018	XP_006352806.1	StTIR1a LP	GAACTGTGGCTGGACGAAGA
		StTIR1a RP	GCGCTAATACCTGCCCATCT
LOC102578728	XP_006359432.1	StTIR1b LP	CTTCAGCTCCCACACACTCC
		StTIR1b RP	ACTGTACGGAAATGGGGTGC
LOC102578999	XP_006362983.1	StTIR1c LP	TCACACGCTTCCCTCAACTC
		StTIR1c RP	GAAGATAGGCACCCCATCCG
LOC102578575	XP_006338135.1	StAFB4 LP	TGTGACGAAGGACTTGAGGC
		StAFB4 RP	ACCTGCTTCAGAAACTGGGG
LOC102595865	XP_006347140.1	StAFB6 LP	AACGTCACCTTGCCTCAGTT
		StAFB6 RP	TGCATACTGCCGACTTGTGA
LOC102590158	XP_006342026.1	SARF5 LP	TGATCTTCCAACAGCGAGTG
		StARF5 RP	ATGAGGAATGAGCCATGGAG
LOC102606235	XP_006357892.1	StARF6a LP	ATATCTCAGGCGCAGATGCT
		StARF6a RP	TGCGAGTTCACTTCTTGGTG
LOC102598513	XP_006350451.1	StARF8b LP	ACATGGTGGCTTTTCTGTCC
		StARF8b RP	TGGCGCTAACAAACACACTC
LOC102594021	XP 006353789.1	StARF19a LP	TGCCCAGCCAAATCAGCATA
		StARF19a RP	ACTGGGTTGTTTCATCCGCT
LOC102582348	XP_006341482.1	StARF2a LP	ACCTTTAGGGAGGCCTGAGT
		StARF2a RP	CCAGGAGCTTTTGAGCCAGA
LOC102586095	XP_006339723.1	StARF18a LP	GTTCTTGCCACTGCATCTCA
		StARF18a RP	CATGACTCACAGCCTCCAGA
LOC102602099	XP_006340145.1	StARF4 LP	TGATAAACCAGTCCGCCCAC
		StARF4 RP	AGGTTTTCCCCAAGCACCAA
102586718/IAA2	NP_001275031.1	StIAA2 LP	GGTCCCAAGGAATGATAGA
		StIAA2 RP	AGTCCAATGGCTTCTGATC
102577631/IAA3	NM_001275398.1	StIAA3 LP	ATTGTGGGACTCAAGGATT
		StIAA3 RP	ACAAACATCTCCCAAGGTA
LOC102595402	XP 006343091.1	StIAA8 LP	GGATGCTAGTCGGCGATGTT
		StIAA8 RP	TCACAGGACATACCTAGCCCA
LOC102593208	XP 006362195.1	StIAA23 LP	TGGAGATTGGATGCTTGTTGG
		StIAA23 RP	TAGCTCTTGGTGCAAGTCCA
LOC102577929	XP 006353472.1	StIAA25 LP	GTGATTGGATGCTTGTCGGTG
		StIAA25 RP	TGGCTCTTGGAGCTAGACCAT
LOC102586390	XP_006350661.1	StIAA12 LP	TCATCATGTAAGAGGCTAA
		StIAA12 RP	CACTTCCCAATCAGTGTAA
LOC102590668	XP_006352755.1	StIAA15 LP	TAGAAGCGAGCTACGTTAA
		StIAA15 RP	CATATCCCAAGGTACATCA
LOC102602619	XM_006352729.2	StABP1 LP	ACTGGTCAATGCCACACACA
		StABP1 RP	AGCTCGTCTTTCCTCGTTGTT
LOC102605763	XM_006350898.2	StTMK1 LP	ATGCCTTTACTGGTCCCG
		StTMK1 RP	CCTTTGAACACCGGCATTGG
LOC102590845	XM_006364198.2	StSPK1 LP	TCGCAGTAAGGCAAAAGGGT
		StSPK1 RP	TGCTCCAGTGCCATTGACTT
LOC102579014	XM_015312788.1	StSKP2a LP	CTCGATCTGTGTGGCTGTGT
		StSKP2aRP	TAGGCCGAGGGATCTCAAGT
LOC102601775	XM_006341910.2	StMPK14 LP	ACTGCTTGTGATGTCTATCGGAA
		StMPK14 RP	AATGAGATGTGGGGAGGGTG
LOC102581018	XM_015306170.1	StTPL LP	AGCAAAGATGGGGAATCGCA
		StTPL RP	CACCCAGCGATCTTTTCCGA

Supplementary figures

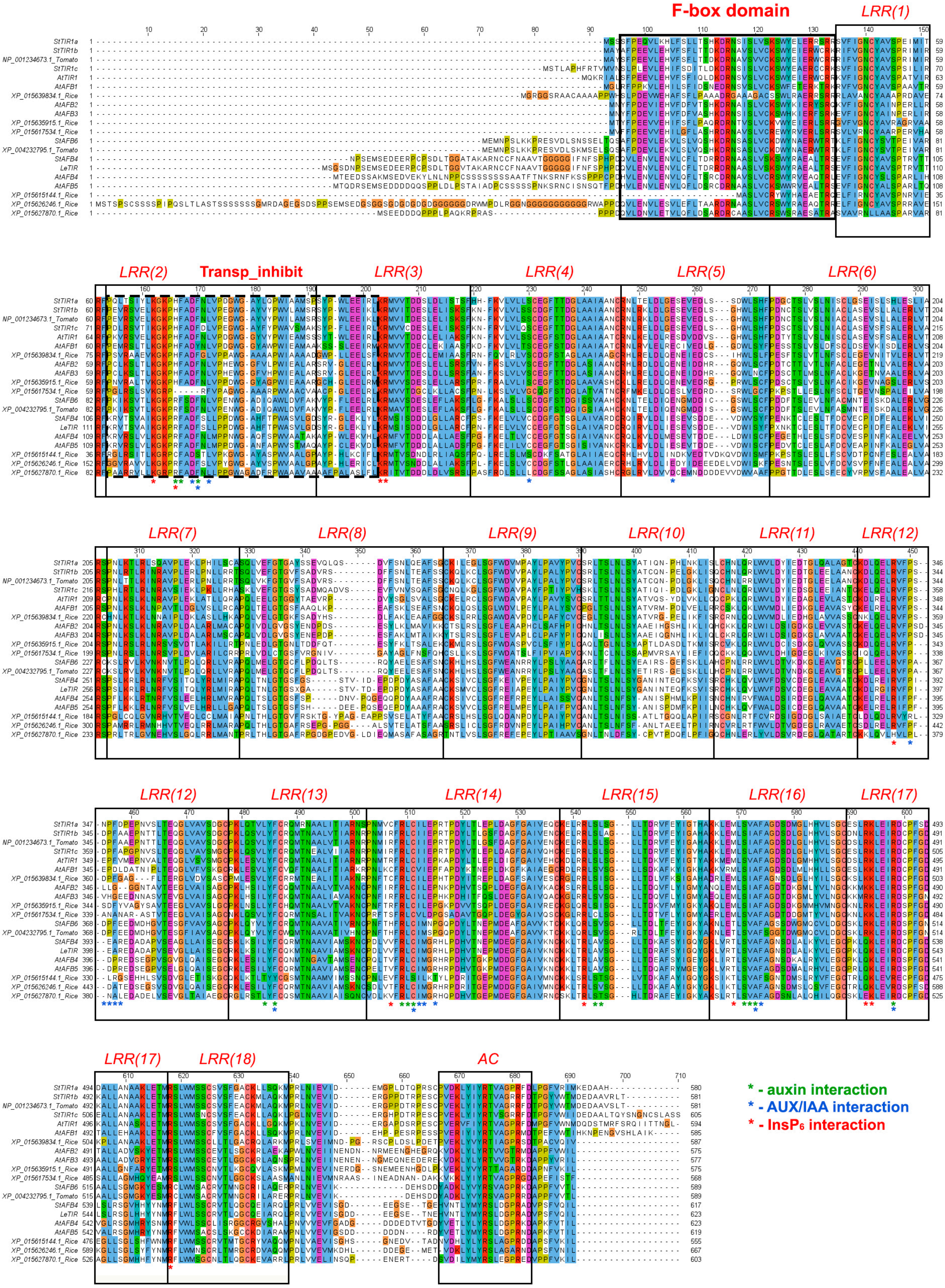

Figure S1. TIR1/AFB ortholog sequence alignment.

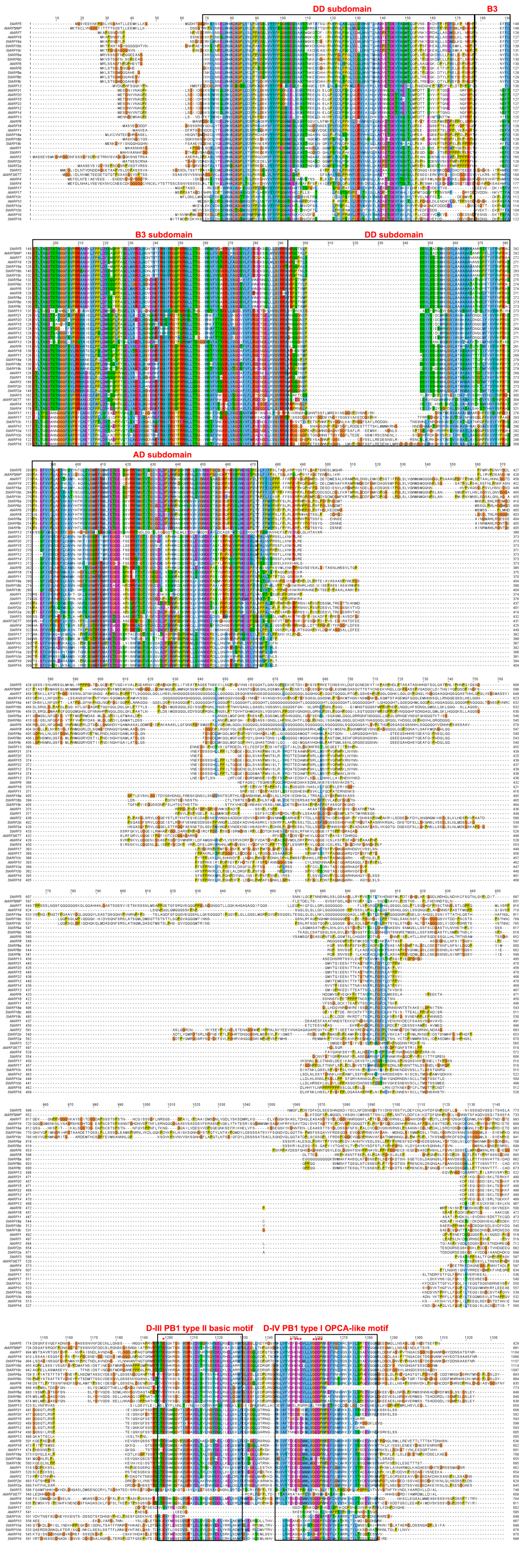

Figure S2. ARF ortholog sequence alignment.

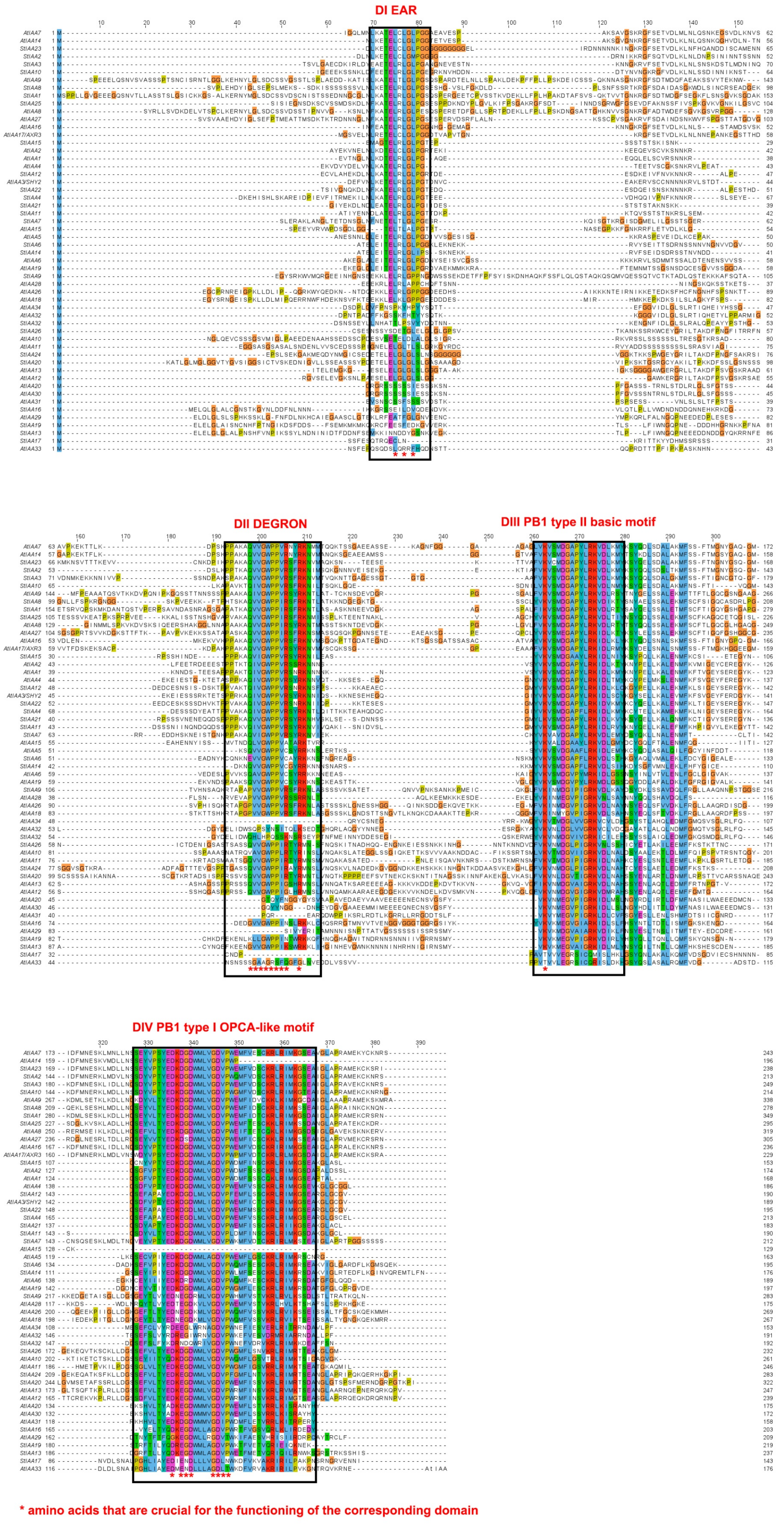

Figure S3. Aux/IAA ortholog sequence alignment.

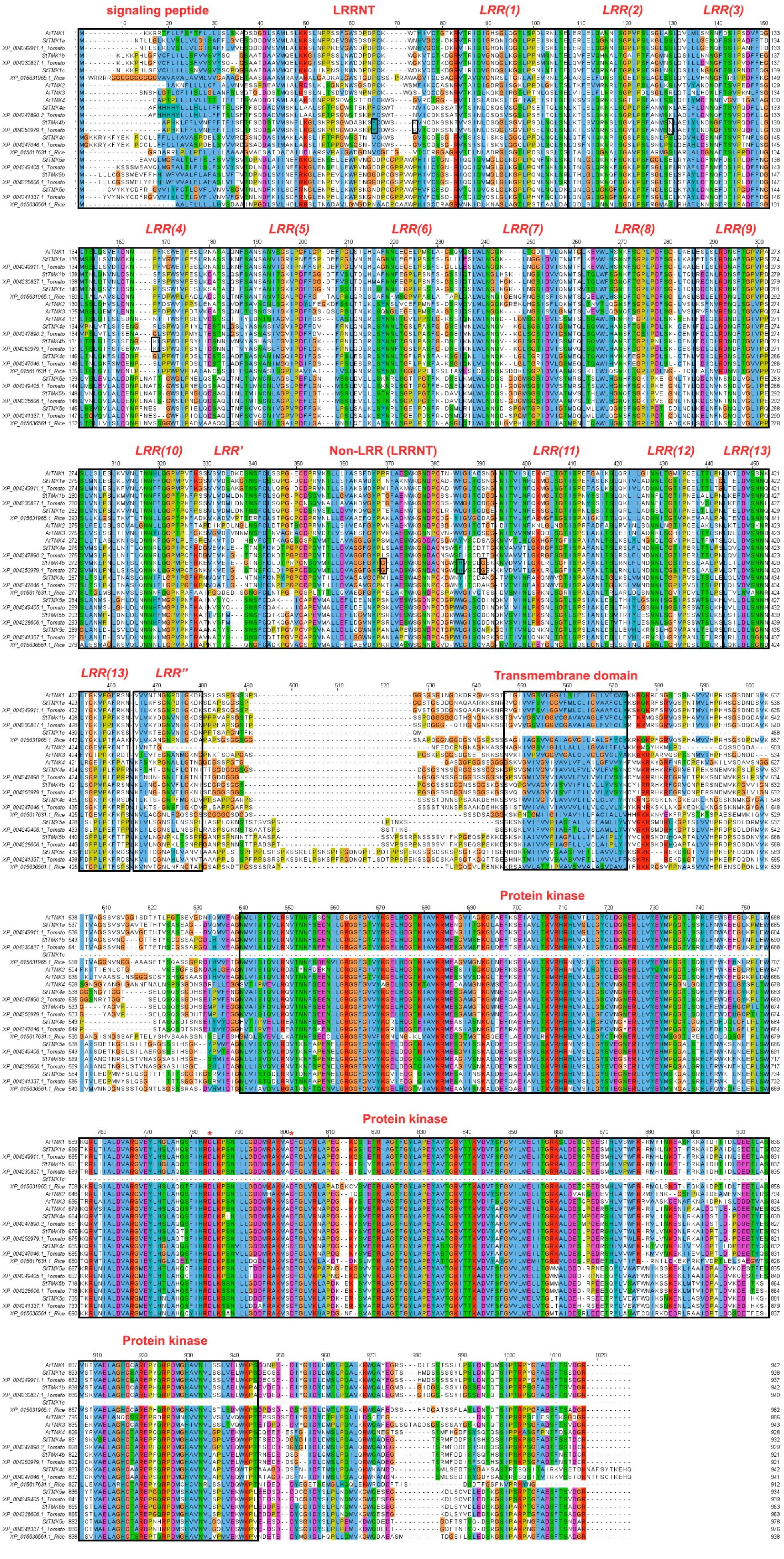

Figure S4. TMK ortholog sequence alignment.

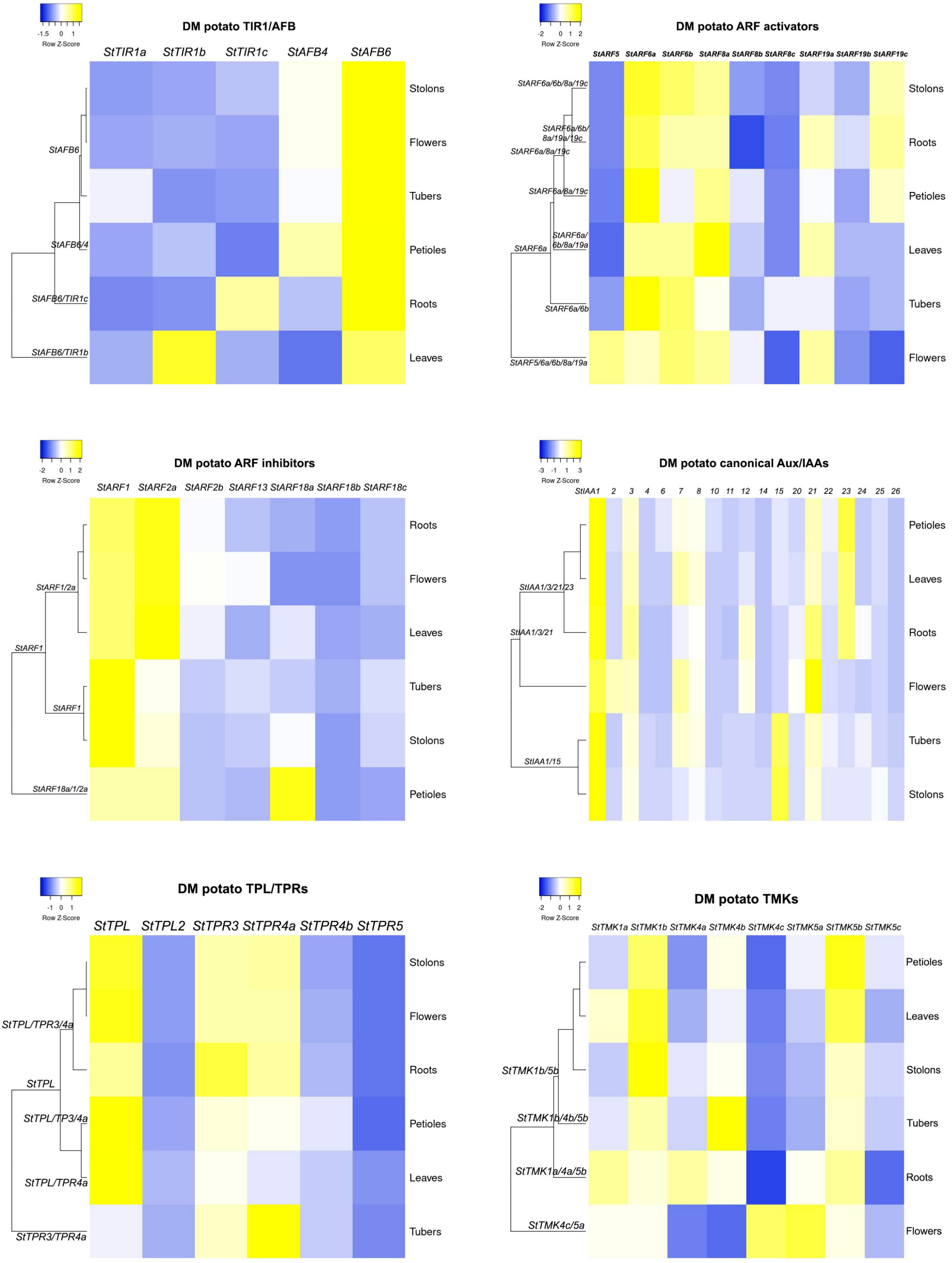

Figure S5. Cluster analysis of DM genes expression (described in Methods). Relative expression data are given by row. Yellow corresponds to high activity, blue to low activity. Branches characterized by preferential expression of the corresponding signaling components are signed.

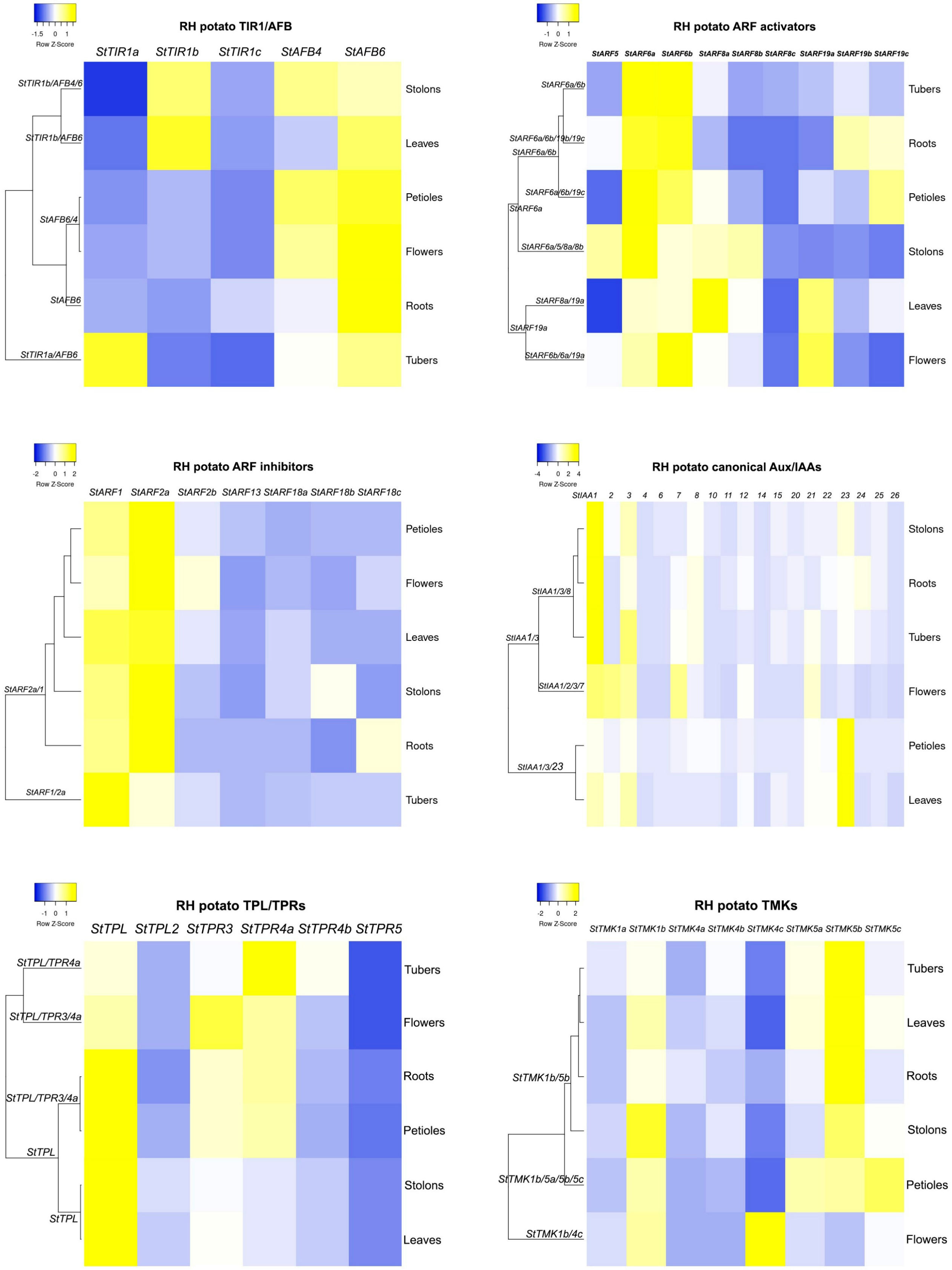

Figure S6. Cluster analysis of RH genes expression (see the legend to Figure S5 for details).

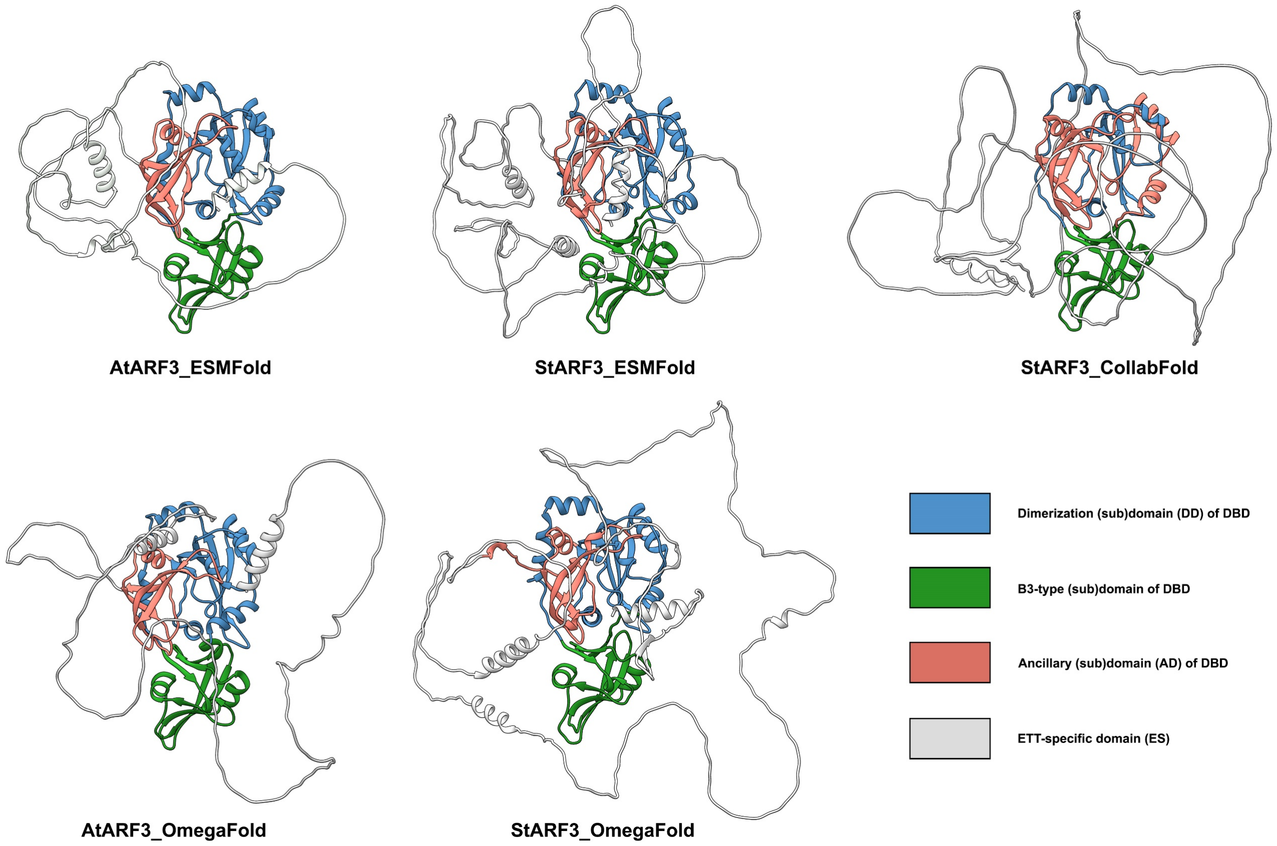

Figure S7. Results of *de novo* modeling of full-size *A. thaliana* AtARF3 and *S. tuberosum* StARF3 ETTs using various services implemented in COSMIC^2 [101]: ESMFold, OmegaFold and CollabFold. Red, blue and green indicate the various subdomains of the DBD domain, gray - ET-specific domain (ES). The short unfolded fragments at the N-terminus of the DBD domain have been removed, for better visibility of the C-terminal ES domain.

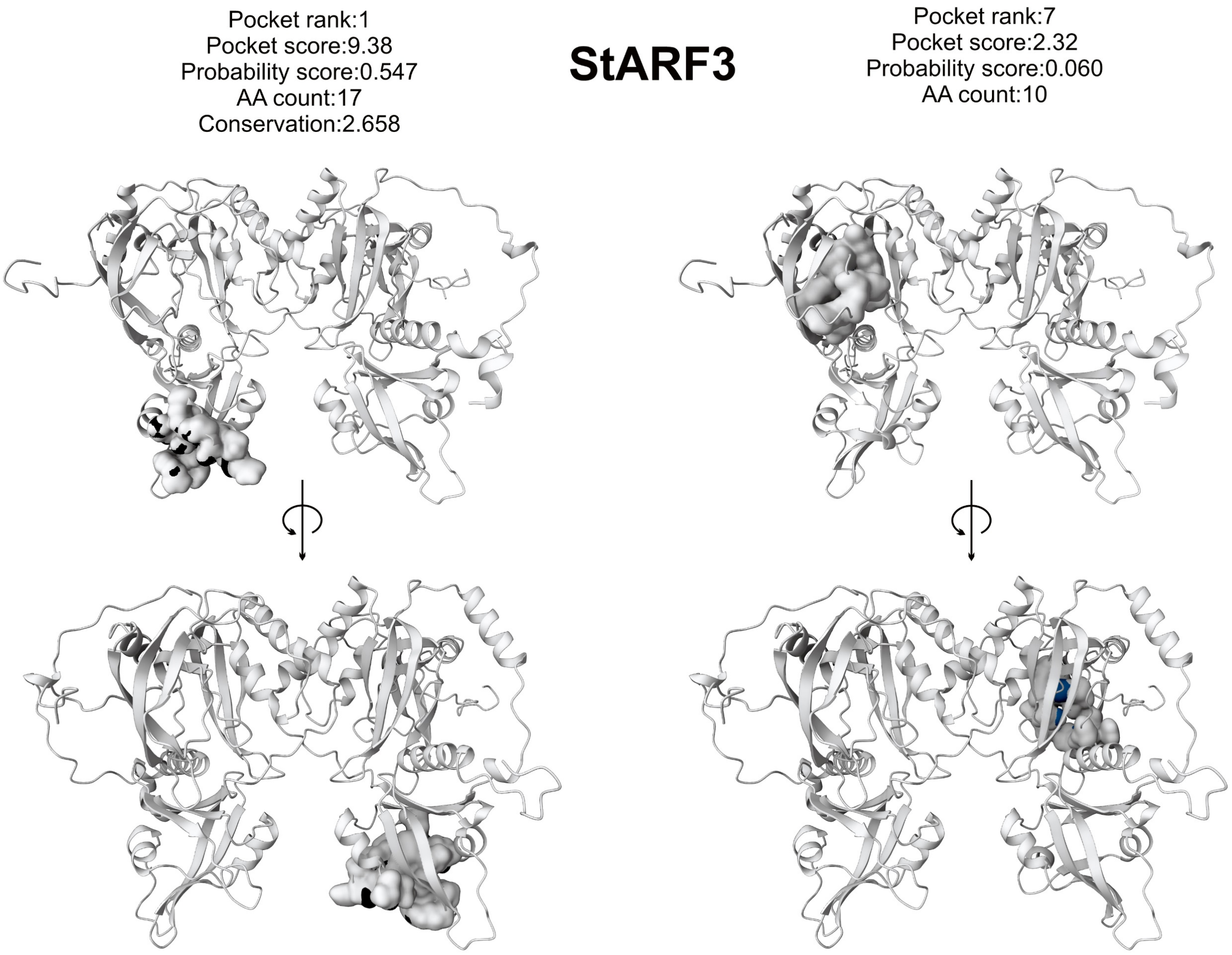

Figure S8. Prediction of possible binding sites for a potential noncanonical signaling member protein StARF3, in the PrankWeb server. On the left is the best result; on the right is the result that topologically corresponds to the binding site of the TRIS molecule in the crystal structure of AtARF1 (PDB ID: 6YCQ) [88]. Models are presented in two projections.
